# Increased spatial coupling of integrin and collagen IV in the immunoresistant clear-cell renal-cell carcinoma tumor microenvironment

**DOI:** 10.1186/s13059-024-03435-z

**Published:** 2024-12-05

**Authors:** Alex C. Soupir, Mitchell T. Hayes, Taylor C. Peak, Oscar Ospina, Nicholas H. Chakiryan, Anders E. Berglund, Paul A. Stewart, Jonathan Nguyen, Carlos Moran Segura, Natasha L. Francis, Paola M. Ramos Echevarria, Jad Chahoud, Roger Li, Kenneth Y. Tsai, Jodi A. Balasi, Yamila Caraballo Peres, Jasreman Dhillon, Lindsey A. Martinez, Warren E. Gloria, Nathan Schurman, Sean Kim, Mark Gregory, James Mulé, Brooke L. Fridley, Brandon J. Manley

**Affiliations:** 1 Department of Biostatistics and Bioinformatics, Moffitt Cancer Center, Tampa, FL 33612 USA; 2 Department of Genitourinary Oncology, Moffitt Cancer Center, Tampa, FL 33612 USA; 3 Department of Urology, Oregon Health & Science University, Portland, OR 97239 USA; 4 Department of Immunology, Moffitt Cancer Center, Tampa, FL 33612 USA; 5 Department of Pathology, Moffitt Cancer Center, Tampa, FL 33612 USA; 6 Tissue Core, Moffitt Cancer Center, Tampa, FL 33612 USA; 7 Nontherapeutic Research Operations, Moffitt Cancer Center, Tampa, FL 33612 USA; 8 NanoString, Seattle, WA 98109 USA; 9 Division of Health Services and Outcomes Research, Children’s Mercy Hospital, Kansas, MO USA

**Keywords:** Single-cell RNA, Malignant-cell typing, Immunotherapy resistance, Spatial transcriptomics, Ligand receptor

## Abstract

**Background:**

Immunotherapy has improved survival for patients with advanced clear cell renal cell carcinoma (ccRCC), but resistance to therapy develops in most patients. We use cellular-resolution spatial transcriptomics in patients with immunotherapy naïve and exposed primary ccRCC tumors to better understand immunotherapy resistance.

**Results:**

Spatial molecular imaging of tumor and adjacent stroma samples from 21 tumors suggests that viable tumors following immunotherapy harbor more stromal CD8 + T cells and neutrophils than immunotherapy naïve tumors. *YES1* is significantly upregulated in immunotherapy exposed tumor cells. Spatial GSEA shows that the epithelial-mesenchymal transition pathway is spatially enriched and the associated ligand-receptor transcript pair *COL4A1*-*ITGAV* has significantly higher autocorrelation in the stroma after exposure to immunotherapy. More integrin αV + cells are observed in immunotherapy exposed stroma on multiplex immunofluorescence validation. Compared to other cancers in TCGA, ccRCC tumors have the highest expression of both *COL4A1* and *ITGAV*. Assessing bulk RNA expression and proteomic correlates in CPTAC databases reveals that collagen IV protein is more abundant in advanced stages of disease.

**Conclusions:**

Spatial transcriptomics of samples of 3 patient cohorts with cRCC tumors indicates that COL4A1 and ITGAV are more autocorrelated in immunotherapy-exposed stroma compared to immunotherapy-naïve tumors, with high expression among fibroblasts, tumor cells, and endothelium. Further research is needed to understand changes in the ccRCC tumor immune microenvironment and explore potential therapeutic role of integrin after immunotherapy treatment.

**Supplementary Information:**

The online version contains supplementary material available at 10.1186/s13059-024-03435-z.

## Background

Using immunotherapy (IO) for treating clear-cell renal-cell carcinoma (ccRCC) has improved response rates and survival outcomes compared to those rates and outcomes of the targeted-therapy era [1–3]. However, IO resistance limits progression-free survival to 11 to 15 months [1, 2]. Certain histological features of ccRCC, such as sarcomatoid morphology, portend an excellent response to IO treatment [4]. Yet reliable biomarkers of IO responsiveness or resistance in other cancers have not been shown to translate to ccRCC [5]. Thus, there is a dire clinical need to discover distinct mechanisms that stratify IO-responsive from IO-resistant phenotypes.

Elements of the tumor immune microenvironment (TIME) have been found to fluctuate as patients with ccRCC progress [6, 7]. Large-scale efforts to characterize the ccRCC TIME through bulk transcriptomic sequencing elucidated the macro-level immune-cell composition within ccRCC [8, 9]. These studies have revealed the association of M2-macrophage infiltration, T-cell exhaustion, and angiogenesis-enriched molecular profiles with survival outcomes and treatment responses in both the tyrosine kinase inhibitor (TKI) and IO eras. Advances in single-cell RNA sequencing (scRNA-seq) allowed profiling of heterogenous cell populations within tumor and surrounding *normal* tissue [10]. More recently this technology has been used to identify differences in various T-cell populations between IO-responsive and IO-resistant tumors.^11^ In these ways, scRNA-seq has deepened our understanding of various cell lineages among various ccRCC tumor stages; however, many questions remain regarding the biology of cell-to-cell interactions across the clinical spectrum in ccRCC. A limitation of scRNA-seq is that the tissue is disassociated and thus does not preserve the tissue architecture (i.e., removes the spatial locations and relationships between individual cells).

Technological advancements may overcome some of the disadvantages of bulk and single-cell methods. Specifically, spatial proteomics has identified the association of certain myeloid-cell line clustering within tumoral regions and poor treatment response with IO, as well as survival outcomes [6, 11, 12]. These studies used spot-based or mini-bulk resolution, but this resolution does not elucidate cell-to-cell crosstalk. Recently, high-plex spatial transcriptomics has allowed simultaneous analysis of cell-level variations in gene expression on a single slide. In situ hybridization of RNA transcripts in formalin-fixed specimens can yield subcellular spatial information and recapitulates the signals seen in bulk RNA sequencing and scRNA-seq [13]. On some platforms, this allows investigation of nearly 1000 transcripts among millions of cells on the same slide [14].

Using the NanoString CosMx Spatial Molecular Imager (SMI) for spatial transcriptomic analysis [14], we evaluated the TIME in 3 clinically relevant patient populations and the TIME of their ccRCC tumors: (1) those who had yet to undergo IO treatment and had tumors without sarcomatoid features (i.e., IO-naïve nonsarcomatoid ccRCC tumors); (2) those who had yet to undergo IO treatment and had tumors with sarcomatoid features (i.e., IO-naïve sarcomatoid ccRCC tumors); and (3) and those treated with IO (i.e., IO-exposed ccRCC tumors). These 3 patient cohorts represent clinically diverse outcomes, with patients’ tumors that are IO naïve and patients’ tumors with sarcomatoid features having potentially enhanced initial response to IO treatment. Residual tumors present after IO treatment have sustained eco-evolutionary selective pressures and subsequently contain subpopulations of IO-resistant tumor cells that harbor the genomic potential for clinical progression [15, 16]. Thus, our study objective was to define the unique cellular and spatial characteristics of these clinically relevant ccRCC cohorts using high-resolution spatial transcriptomic technology.

## Results

### Quality control

Each patient-tumor sample had a 1-mm sample obtained from a region of tumor and stroma near the respective tumor-stroma interface. SMI was obtained on 3 tissue microarrays (TMAs) using the Human Immuno-Oncology Panel (consisting of 978 RNA probes). Between stromal and tumor primary-disease fields of view (FOV), 40 FOV passed the applied quality filters (see Methods, Table 1, Additional File 1: Fig. S1, Additional File 2: Table S1. The number of cells on the remaining FOV ranged from 930 to 6090, with a median of 2969 cells per FOV.

**Table 1:** Patient cohorts for this study

	*N*
Patients	21
Samples	
Tumor	18
Stroma	21
Treatments (patients)	
Ipi-Nivo	2 (4)
Pembo-Axi	4 (7)
None	15 (28)
Sarcomatoid: IO naïve	
No	8 (14)
Yes	7 (14)
Median Survival (months)*	
Pre-IO Non-Sarcomatoid	69.5
Pre-IO Sarcomatoid	29.9
Post-IO	15.2

^*^Days divided by (365.25/12)

### Subclustering T cells and mononucleic phagocytes (MNPs) identify more refined cell types

**Fig. 1:**
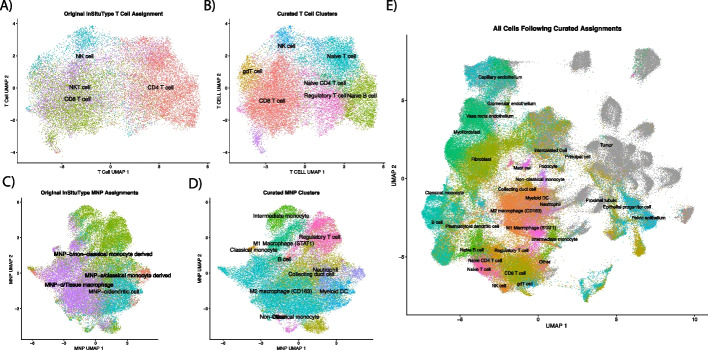
Phenotyping of cells following assignment with *InSituType*. **A** and **C** show UMAPs of T cells and MNPs, respectively, calculated with all cells in all FOV. Refined phenotypes from calculating new PCA/UMAP, clustering the subset with Louvain, and identifying markers with “FindAllMarkers” can be seen in **B** and **D**. Final cell assignments of all cells, including tumor cells, are shown in **E**. Abbreviations: FOV, fields of view; MNPs, mononucleic phagocytes; PCA, principal component analysis; UMAP, uniform manifold approximation and projection

Subclustering for T cells (Fig. 1A) with Louvain clustering identified 8 unique clusters from the first 50 principal components (Fig. 1B). Between these 8 unique subclusters, 112 unique genes were identified as differentially expressed in at least 1 cluster (absolute log-fold change [LFC] > 0.25 and *P* < 0.004) (Additional File 2: Table S2). One of the new Louvain clusters was dominated by CD4 + T cells and higher *FOXP3* gene expression, and thus the new cluster was identified as regulatory T cells (Fig. 1B). Subclustering of mononucleic phagocytes (MNPs) resulted in 14 distinct clusters (Fig. 1C, D). Among the 14 clusters, 189 unique genes were found to be differentially expressed (absolute LFC > 0.25 and *P* < 0.004; Additional File 2: Table S2). Seven of those clusters showed signatures of MNPs, while the other 6 were more consistent with regulatory T cells, collecting duct cells (× 3), neutrophils (× 2), and B cells (Additional File 2: Table S2, Fig. 1D). Fig. 1E shows uniform manifold approximation and projection (UMAP) of all cells with their final phenotype assignments.

### Identification of malignant ccRCC cells from normal reference

**Fig. 2:**
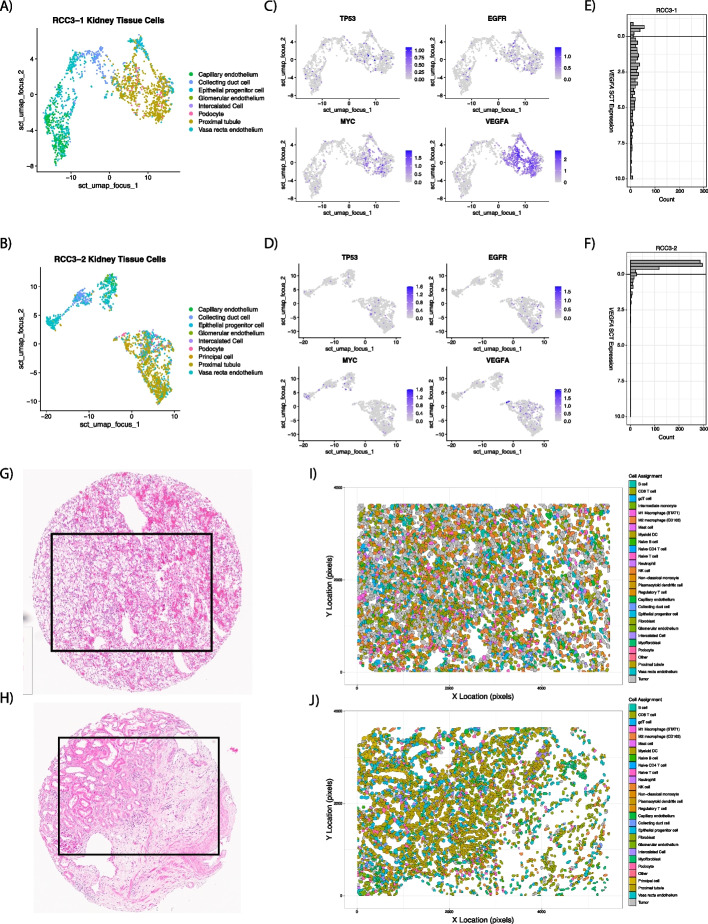
Examples of FOV used for malignant-cell identification with LASSO generalized linear models after cell typing with *InSituType* and the Kidney Cell Atlas. UMAPs were created of kidney tissue specific cells (nonimmune, nonfibroblast) for a tumor (**A**) and stroma (**B**) FOV. Gene expression was plotted over the UMAP for TP53, EGFR, MYC, and VEGFA with “FeaturePlot” to aid in identifying malignant cells (**C** and **D**). VEGFA showed high expression in malignant-proximal tubule cells (**E**), while expression in normal proximal tubule cells (**F**) was low. H&E images (**G** and **H**) who cores sent for CosMx SMI. After *InSituType* and malignant-cell classification, polygon plots were constructed with final cell assignments (**I** and **J**). Abbreviations: FOV, fields of view; LASSO, least absolute shrinkage and selection operator; UMAP, uniform manifold approximation and projection

After cell typing using *InSituType* with the Kidney Cell Atlas, UMAPs were created (Fig. 2A, B) to identify FOV for classifying tumor cells, and gene expression of *TP53*, *EGFR*, *MYC*, and *VEGFA* was plotted (Fig. 2C, D) to identify FOV for classifying normal cells. As anticipated, *VEGFA* was high for proximal tubule cells on tumor FOV (Fig. 2E), while stromal FOV showed lower expression in proximal tubule cells (Fig. 2F). The spatial context of these cells can be further seen in their H&E images (Fig. 2G, H) as well as in the cell-typed polygon plots (Fig. 2I, J).

Differential gene expression was performed to find marker genes specific to either the malignant or normal proximal tubule cells aided by visual correlation on matched H&E images, resulting in 51 differently expressed genes (Additional File 2: Table S3). Gene expression of these 51 genes was passed to a logistic regression model with Least Absolute Shrinkage and Selection Operator (LASSO, tenfold cross-validation) along with the class of the proximal tubule cell (normal or malignant) to reduce the number of genes needed to predict class. This decreased the number of genes from 51 to 43 (Additional File 2: Table S3) to be used within the generalized linear model to predict malignant classification (Fig. 2I, J). Further review of the LASSO predictions by a genitourinary pathologist (JD) led to only one FOV reclassification for downstream analyses to *stroma* (FOV17 on slide RCC4). The average cohort abundance of tumor/stroma is shown in Additional File 1: Fig. S2.

To externally assess our LASSO model’s ability to identify malignant cells, we applied it to another RCC single-cell RNA-Seq (scRNA-seq) dataset from Li et al. [17] All genes in the LASSO model were present except for *WIF1*, which is due to low coverage in the scRNA-seq because it is a whole transcriptome platform. The LASSO model produced an area under the curve of 0.91 against the kidney-specific cell types (renal-cell carcinoma—tumor cells, endothelial cells, proximal tubule epithelial cells, and nonproximal tubule epithelial cells). Setting the threshold for malignant-cell classification the same as our CosMx SMI data (under the predicted value of 0.5 was classified as malignant) showed 12,833 of the 13,105 cells being correctly classified as malignant (97.5%) and 6226 of 8954 correctly classified as nonmalignant (69.5%).

### Higher abundance of CD8 T cells and neutrophils in the *IO*-exposed stromal TIME and M2 macrophages in the sarcomatoid stromal TIME

Within the tumor FOV, there were no differences in phenotype abundances between IO-naïve and IO-exposed tissue samples (Additional File 2: Table S4). However, the cell phenotypes in the stromal FOV showed CD8 + T cells (false discovery rate [FDR] = 0.0007) and neutrophils (FDR = 0.011) significantly higher in the IO-exposed samples compared to IO-naïve samples (Additional File 2: Table S4). Nonmalignant proximal tubule cells were significantly more abundant in IO-naïve FOV (FDR = 0.018), while tumor cell counts were not significantly different between IO-exposed and IO-naïve FOV (FDR = 0.939). Myofibroblasts and fibroblasts were not significantly more abundant in the stromal FOV of IO-naïve than IO-exposed (myofibroblast FDR = 0.637, fibroblast FDR = 0.503).

When comparing IO-naïve samples with and without sarcomatoid features, none of the cell phenotypes showed differences in abundances in tumor FOV (Additional File 2: Table S4). In the stromal FOV, M2 macrophages showed significantly higher abundance in sarcomatoid samples compared to nonsarcomatoid samples (FDR = 0.044, Additional File 2: Table S4).

### Minimal changes in cell-clustering differences between patient cohorts

We used a quantitative framework leveraging Ripley’s* K* estimates, a measure of spatial heterogeneity commonly used in ecology and economics, to identify differences in cell-type spatial clustering [18]. We observed that principal cells in the stroma of those exposed to IO were significantly more clustered than they were in IO-naïve samples (FDR = 0.046; Additional File 2: Table S5). All other cell types showed no differences between IO-naïve and IO-exposed in both tumor and stroma FOV.

The Ripley’s *K* estimates of individual cell types in IO-naïve nonsarcomatoid and sarcomatoid feature tissues did not show significant differences in either tumor or stromal FOV (Additional File 2: Table S5).

### YES1 significantly upregulated in tumor cells following *IO* exposure

Pseudobulk analysis is the aggregated expression of genes over all cell types using single-cell data [19]. On pseudobulk analysis, IO-exposed tumor FOV showed 13 genes significantly downregulated and a single gene with higher expression than IO naïve (*YES1* FDR = 0.084; Additional File 2: Table S6). The most significant downregulated gene in IO-exposed tumor FOV was *TPSB2* (FDR = 0.019).

In our differential expression analysis at the cell phenotype level, tumor cells from IO-exposed tumor FOV showed only 15 genes significantly higher than tumor cells from FOV naïve to IO, and *YES1* was the most significant (FDR = 0.026). In the IO-exposed tumor FOV, principal cells (633 genes) and pelvic epithelium (113 genes) had the largest numbers of genes significantly higher compared to IO-naïve tumor FOV (Additional File 2: Table S7).

**Fig. 3:**
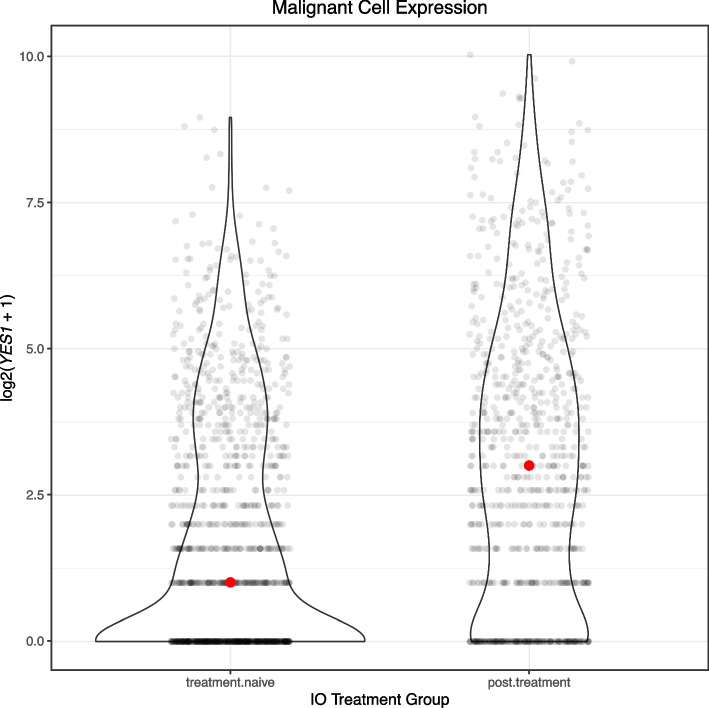
Expression of *YES1* in malignant melanoma cells before and after treatment with immune checkpoint inhibitor anti-PD-1 (GSE115978)

Exploring the melanoma dataset GSE115978 [20], which has before-and-after exposure to IO, we performed a log 2 transformation of cells labeled Mal (Fig. 3). Expression of *YES1* following treatment in malignant cells showed a median log2 expression of 3 (raw count = 7), while median log2 expression treatment in naïve malignant cells was 1 (raw count = 1). A Wilcoxon rank sum test between malignant cells exposed and not exposed to treatment showed that *YES1* expression was significantly higher after exposure to IO (*P* = 1.08E − 32).

On pseudobulk analysis of IO-naïve sarcomatoid vs nonsarcomatoid samples, there were no genes expressed significantly different (Additional File 2: Table S6). In sarcomatoid-tumor FOV, differential expression by individual cell phenotype revealed tumor cells having significant downregulation of *IL17A* compared to nonsarcomatoid-tumor FOV (FDR < 0.001) along with 7 other genes. No genes were significantly upregulated in tumor cells (Additional File 2: Table S8).

### Multiple integrin genes upregulated in IO-exposed cells in the stroma TIME

Among stromal FOV, *GPX3* is the most significant downregulated gene in IO-exposed tumor cells (FDR < 0.001), and *AZU1* was found to be downregulated in regulatory T cells (FDR < 0.001) and fibroblasts (FDR < 0.001; Additional File 2: Table S9). B cells showed the most genes (334) expressed higher in IO-exposed FOV than in IO-naïve FOV, while the following also had high expressions in many genes: vasa recta endothelium (158), fibroblasts (154), tumor (130), and regulatory T cells (123). Among stromal fibroblast cells, *ITGAV*, *ITGA1*, *ITGB1*, and *ITGB5* were significantly upregulated following IO and, notably, isolated tumor cells found in the stroma showed upregulation of *B2M*, *VIM*, *ITGA5*, *ITGB2*, and *ITGA3* (Additional File 2: Table S9).

The most significant changes in expression between sarcomatoid-tumor FOV and nonsarcomatoid-tumor FOV occurred in CD8 T cells, including downregulation of the anti-inflammatory RNA-binding protein *ZFP36* (FDR < 0.001) and downregulation of *TGFBR2* (FDR < 0.001; Additional File 2: Table S8). Two genes (*EPOR* and *LIFR*) were significantly downregulated in stromal M2 macrophages in sarcomatoid tissues, while no genes were significantly upregulated (Additional File 2: Table S10). B cells also showed 8 genes upregulated in sarcomatoid samples, including *COL9A3* and *ITGB2* (FDR < 0.001 for both).

#### Epithelial-mesenchymal transition (EMT) genes are spatially enriched in IO-exposed samples

**Fig. 4:**
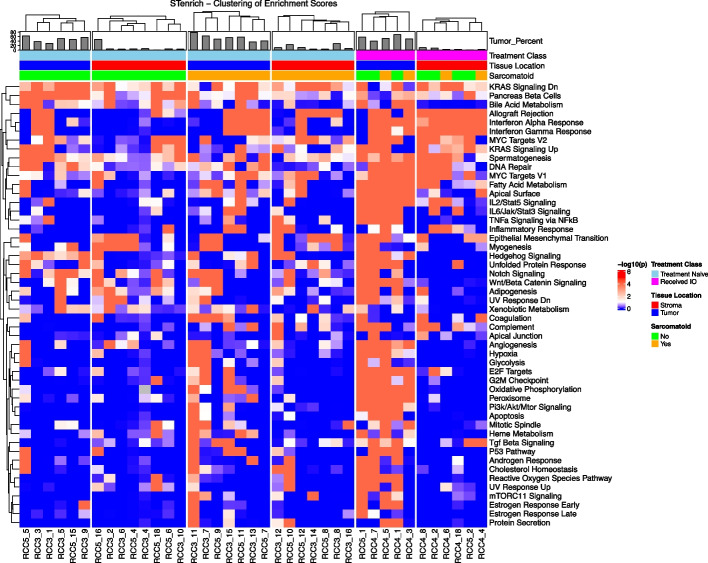
Spatial enrichment of cells with high (> mean + 1 standard deviation) hallmark gene sets scores on tumor and stromal FOV showing high spatial enrichment of gene sets in IO-exposed tumor FOV. Abbreviations: FOV, fields of view; IO, immunotherapy

Tumor FOV exhibited spatial enrichment of the epithelial-mesenchymal transition (EMT) gene set, with spatial aggregation observed in 4 of 5 samples exposed to IO and in only 1 of 6 IO-naïve samples (Additional File 2: Table S11). This is displayed in Fig. 4 in “Tumor Locations” with increasingly significant spatial clustering, moving from Treatment Naïve (left) to Sarcomatoid (middle) to finally those tumors that were IO-exposed (right). Expression of the hypoxia gene set was also spatially clustered in all TKI- and IO-exposed tissue samples; in contrast, only 1 IO-naïve sample showed spatial clustering. The hypoxia gene set was clustered in 1 patient who received ipilimumab/nivolumab (*P* = 0.001). Higher spatial clustering was also seen in IL2/STAT5 signaling (5 of 5 IO-exposed with *P* < 0.001; 1 of 6 IO-naïve with *P* < 0.1); IL6/JAK/STAT3 signaling (4 of 5 IO-exposed with *P* < 0.001; 1 of 6 IO-naïve with *P* < 0.1); MYC targets V1 (3 of 5 IO-exposed with *P* < 0.001; 2 of 6 IO-naïve with *P* ≤ 0.001); and oxidative phosphorylation among IO-exposed samples (5 of 5 IO-exposed with *P* < 0.001; 0 of 6 IO-naïve with *P* < 0.1). Very few gene sets showed more clustering in IO-naïve samples than in samples exposed to IO (Fig. 4).

In stromal FOV, interferon α and gamma response were clustered in all IO-exposed samples, with no clustering observed in IO-naïve samples (Additional File 2: Table S11). Both IO-naïve and IO-exposed groups harbored samples with cells that have high enrichment of EMT clustering.

In sarcomatoid-tumor FOV, TGF-β signaling was spatially enriched in 3 of 7 sarcomatoid samples, and in none of the nonsarcomatoid samples. Genes related to the G2M checkpoint displayed significant spatial enrichment in 3 sarcomatoid samples and in none of those samples without sarcomatoid features. Also, 4 sarcomatoid samples showed spatial enrichment of oxidative phosphorylation, while no nonsarcomatoid showed spatial enrichment (Additional File 2: Table S11).

In sarcomatoid stromal FOV, TNF-α signaling via NFKβ showed significant spatial enrichment in 3 of 7 sarcomatoid stromal samples and in 0 of 8 nonsarcomatoid stroma samples along with the complement gene set (Additional File 2: Table S11). Genes involved in adipogenesis demonstrated significant spatial enrichment in 5 of the 8 nonsarcomatoid samples and in 2 of the 7 nonsarcomatoid samples (Fig. 4).

### Ligand receptors COL4A1 and ITGAV spatially autocorrelated in the stroma of IO-exposed samples

Moran’s *I* is a statistical measure that assesses spatial autocorrelation between 2 variables, which is the degree to which high or low values of those 2 variables correlate across a Euclidean distance. An FOV in which 2 genes are highly expressed between many neighboring cells will generate an elevated bivariate Moran’s *I* (where values range from − 1 to + 1, with negative values indicating an inverse spatial relationship and positive values indicating a direct spatial relationship in gene expression). Moran’s *I* for ligand-receptor-pair genes from the EMT gene set were not significantly different between IO-naïve and IO-exposed samples in tumor FOV (Additional File 2: Table S12). The smallest FDR observed was between *TGFB1* and *ITGB5* (FDR = 0.91), where IO-naïve samples averaged a slightly negative Moran’s *I* (*I* = − 0.019), and IO-exposed samples averaged slightly positive Moran’s *I* (*I* = 0.05). Ligand-receptor genes in the IL6/JAK/STAT3 signaling gene set did not show strong differences in spatial autocorrelation between IO-naïve and IO-exposed samples (Additional File 2: Table S13). The level of autocorrelation between the *TGFB1* and *ACVRL1* pair was the most different between IO-naïve and IO-exposed cohorts, with FDR = 0.41.

Among the stromal FOV, the *COL4A1* and *ITGAV* pair showed significant differences in Moran’s *I* between IO naïve and exposed samples (FDR = 0.056; Additional File 2: Table S12). IO-naïve samples had an average Moran’s *I* = 0.0127, while those exposed to IO showed an average Moran’s *I* = 0.120, showing significantly higher spatial autocorrelation following exposure to IO treatment. Interestingly, *ITGAV* expression did not significantly differ between tumor cells in the stroma FOV (FDR = 0.934) while *COL4A1* shows a slightly higher expression following IO exposure compared to IO naïve FOV (FDR = 0.023; Supplementary Tables 9, 12).

Comparing non-sarcomatoid to sarcomatoid samples, bivariate Moran’s *I* did not identify any ligand-receptor pairs from either EMT or IL6/JAK/STAT3 gene sets that were spatially correlated in either the tumor or stroma FOV (Additional File 2: Table S12, 13; Supplemental Text).

#### Fibroblasts, myofibroblasts, and endothelial cells displayed the highest COL4A1 and ITGAV expression.

**Fig. 5:**
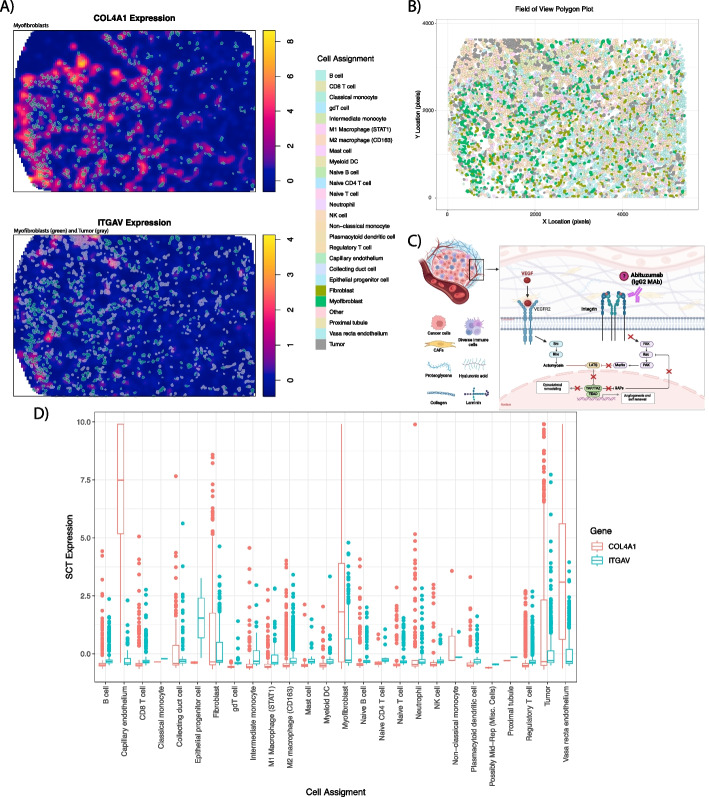
Expression of COL4A1 and ITGAV on RCC4 – FOV8. Figure **A** shows overlap for myofibroblasts and tumor and high gene expression. Locations of all cells and their respective phenotypes are shown in **B**. Pathway description of VEGF mediation neovascularization, of which integrin and YAP are involved (**C**). Expression of *COL4A1* and *ITGAV* for cell types on RCC4 – FOV8 (**D**)

*COL4A1* and *ITGAV* expression was plotted for the FOV with the greatest bivariate Moran’s *I* (RCC4–FOV8) to identify cells associated with regions of high expression (Fig. 5A). Myofibroblasts showed high overlap with regions of high *COL4A1* expression, and both myofibroblasts and tumor cells showed overlap with the areas of high *ITGAV* expression. Locations of all cells in the FOV are shown in Fig. 5B. Boxplots were also constructed to link cellular phenotypes with the greatest expressions of *COL4A1* and *ITGAV*. This showed that fibroblasts, myofibroblasts, and kidney-specific cell types (like tumor cells and endothelium) had the highest expression (Fig. 5D). Notably, tumor cells showed high expressions of both *COL4A1* and *ITGAV*.

#### More fibroblasts express integrin αV in IO-exposed samples on multiplex immunofluorescence (mIF)

**Fig. 6:**
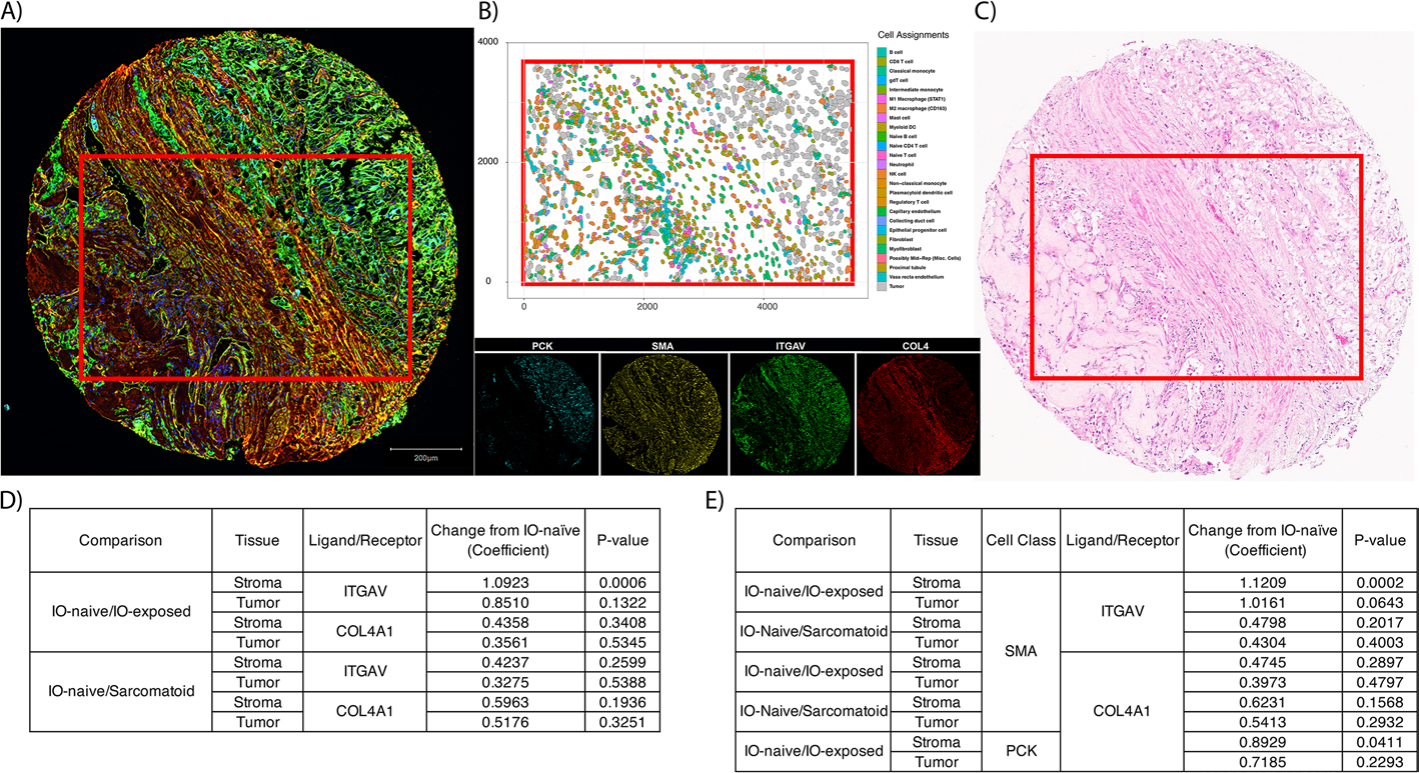
Example core that subjected to different assays. **A** Displays multiplex immunofluorescence staining for pancytokeratin (endothelium, PCK), smooth-muscle actin (fibroblasts, SMA), integrin subunit alpha (ITGAV), and type 4 collagen (COL4). Cell types derived from CosMx SMI gene expression (**B**) show structure identified in both the multiplex immunofluorescence image and H&E (**C**). Abbreviations: H&E, hematoxylin and eosin; PCK, pancytokeratin; SMA, smooth muscle actin; SMI, spatial molecular imager. **D** and **E** show results for comparisons that were made between cohorts at the ligand/receptor assignment level and the fibroblast/tumor + ligand/receptor assignment levels, respectively

To validate our findings of ligand-receptor gene expression in protein products, we conducted multiplex immunofluorescence (mIF) on orthogonal sections of the spatial transcriptomics TMA cores (Fig. 6A). Figs. 6B, C the same core as it was profiled with CosMx SMI as well as the H&E, respectively. Abundances of integrin αV and type IV collagen α1, translational protein products of *ITGAV* and *COL4A1* respectively, were compared between tissue groups (e.g., IO naïve vs IO exposed, sarcomatoid vs nonsarcomatoid) using beta-binomial modeling. We did not identify differences in the percentages of cells that were integrin αV and type IV collagen α1–positive on tumor FOV between IO-naïve and IO-exposed nor between primary IO-naïve and sarcomatoid samples. However, as observed with the IO-naïve vs IO-exposed bivariate Moran’s *I* analysis using gene expression, integrin αV–positive cells on mIF were significantly more abundant after exposure to IO in the stroma as compared to IO-naïve tissues (*P* = 0.001; Fig. 6D; Additional File 2: Table S14).

Next, we set out to determine which cell type was contributing to the difference of integrin subunit αV protein in the IO-exposed samples. IO-naïve and IO-exposed samples showed a significant difference in fibroblast (smooth-muscle actin– [SMA]- marker) cells that are integrin αV–positive in the stromal compartment (*P* < 0.001, Fig. 6E; Additional File 2: Table S15). A similar trend was identified in the tumor compartment (*P* = 0.064).

Compared to IO-naïve samples, the proportion of the area positively stained for type IV collagen α1 (including extracellular protein) was higher among IO-exposed samples in both tumor and stromal regions, but this was not statistically significant (*P* = 0.9 and *P* = 0.4).

#### Ligand receptors COL4A1 and ITGAV have the highest expression in kidney *cancer* compared to other cancers

**Fig. 7:**
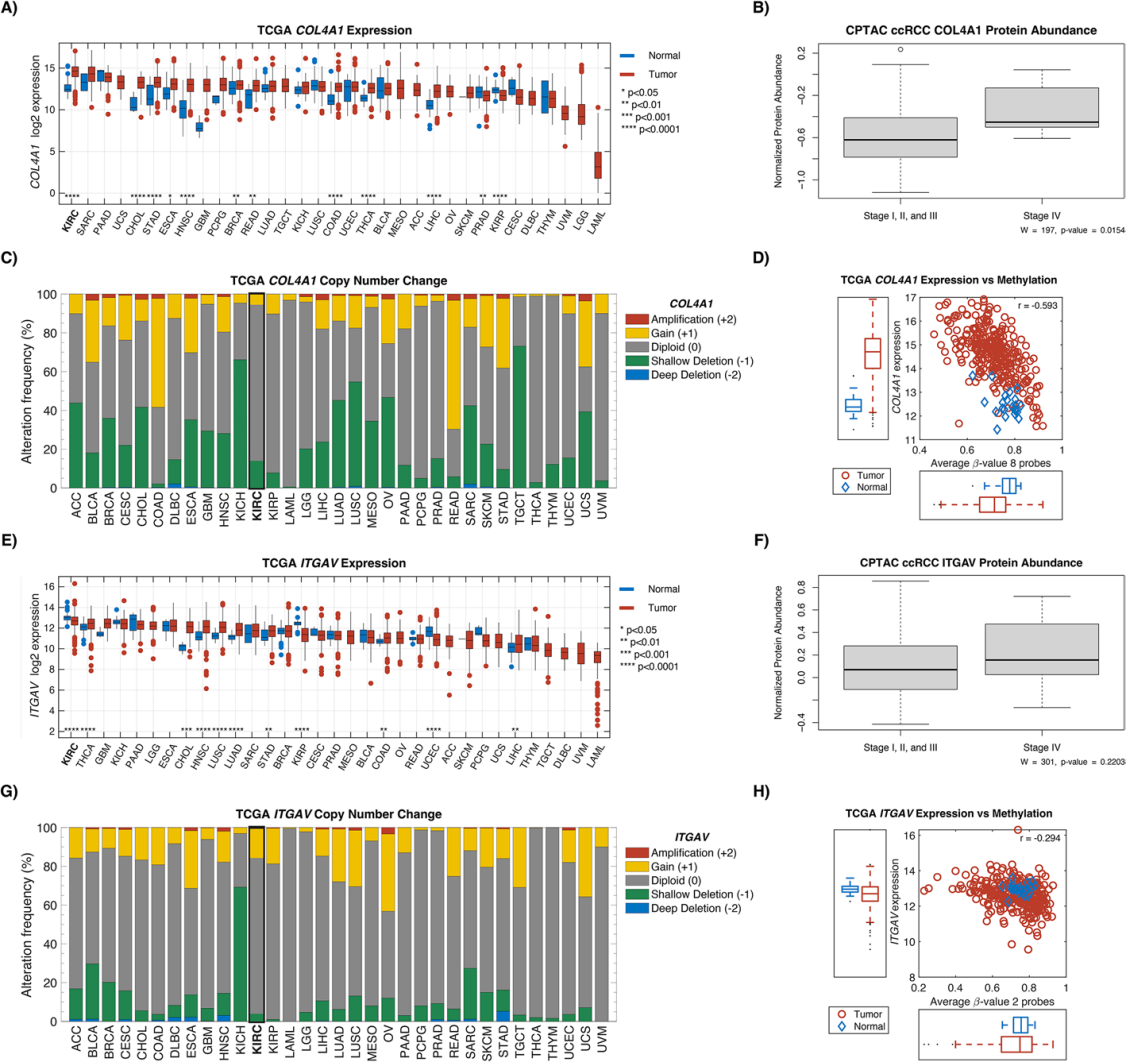
Exploration of COL4A1 and ITGAV in TCGA and CPTAC. Gene expression in tumor and normal for COL4A1 (**A**) and ITGAV (**E**). Protein abundance between low-stage ccRCC (I, II, and III) and high-stage ccRCC (IV) for COL4A1 (**B**) and ITGAV (**F**). **C** and **G** show the copy-number change associated with the 2 genes. TCGA gene expression against methylation levels are shown in **D** and **H**. Abbreviations: CPTAC, Clinical Proteomic Tumor Analysis Consortium; TGCA, The Cancer Genome Atlas

To further characterize the presence of *COL4A1* and *ITGAV* in ccRCC, we examined the Clinical Proteomic Tumor Analysis Consortium (CPTAC), The Cancer Genome Atlas (TCGA), and the Genotype-Tissue Expression (GTEx) project [21, 22]. Examining normal tissue samples from GTEx, we found that kidney and fibroblast cells demonstrated some of the highest expression of these genes compared to other cell types (Additional File 1: Fig. S3). In proteomic analysis from CPTAC, type IV collagen protein expression was significantly higher in advanced ccRCC disease (stage IV) compared to localized disease (stages I–III vs IV, *P* = 0.015; Fig. 7).

Using the PanCan Atlas from TCGA, expression of *ITGAV* and *COL4A1* transcripts was found to be the highest in ccRCC specifically, compared to all other cancers. The vast majority of copy-number variations for these genes were diploid. Interestingly, the *COL4A1* gene was hypomethylated in ccRCC tumor samples compared to adjacent normal tissue (Fig. 7).

## Discussion

Given the lack of clinically validated biomarkers in predicting ccRCC IO efficacy, the cell diversity and spatial heterogeneity of the TIME holds much promise in extracting clinically meaningful information from biopsy or surgical tumor samples. By leveraging differences between treatment naïve and eco-evolutionarily selected residual tumors following IO therapy, we sought to reveal unique characteristics of the IO-resistant TIME using cellular-resolution spatial transcriptomics. Results generated that most cell-abundance and gene-expression changes occur within the stroma compartment of the tumor tissue among immune cells. Spatial gene-set enrichment analysis (GSEA) at cellular resolution revealed enrichment of EMT and IL6 hallmark gene sets. Uniquely, we identified an increase in the stromal autocorrelation of *ITGAV* and *COL4A1* transcripts moving from the IO-naïve to IO-exposed setting which represents a unique spatial relationship not previously identified in ccRCC. Finally, we validated these associations at the protein level through multiplexed immunofluorescence.

High EMT enrichment scores on bulk RNA sequencing are associated with worse disease-specific survival in ccRCC [23], and various EMT ligand-receptor pairs have been implicated [24–26]. Prior spatial transcriptomic work using spot-based resolution has also identified EMT-rich tumor cells being associated with worse prognosis in the TCGA cohort [17]. Still, specific ligand-receptor pairs from the EMT pathway were not elucidated in these studies. This may reflect the limitation of scRNA-seq data or, in the case of spot-based spatial transcriptomics, the loss of cell-to-cell spatial information. Although average ligand expression is high in one cell type and receptor expression is high in another, this does not demonstrate colocalization and the affinity for biologic crosstalk. In this study, we describe a GSEA method that also incorporates spatial data. We hypothesize that the significance of spatial gene-set enrichment represents downstream activation of cell-signaling molecules. Within specific gene sets, we were able to identify a curated list of known ligand-receptor pairs and identify one such signaling pair, which was also spatially enriched and unique to IO-exposed tumors.

EMT activation may confer resistance to therapy in cancer cells by a number of biological pathways, including cell-cycle arrest, alteration of cellular transporters, and dampening the cytolytic activities of CD8 + T cells [27]. *YES1*, the most significantly upregulated gene among IO-exposed tumor cells in this cohort, may explain one specific mechanism via YAP1 (YES-associated protein), which is also associated with poor survival in treatment of treatment naïve ccRCC patients [28, 29]. In human ccRCC cell lines, Chen et al. demonstrated EMT enrichment in cancer cells with upregulated *YAP1*. [30] Given our findings of the upregulation of *YES1* in IO-exposed tumor cells, this remains a plausible mechanism in our cohort. Moreover, the Hippo-YAP pathway is a known linchpin in therapy resistance for many cancers and multiple major drug classes, including immunotherapy [31]. YAP1-mediated immune-resistance is possibly conferred by increased expression of PD-L1 in other cancers. *CD274* expression (gene for PD-L1) was not significantly higher among tumor cells in this study; however, PD-L1 has also not been shown to be a reliable biomarker for immunotherapy response in ccRCC [32]. Thus, additional studies of *YAP1* and related Hippo-signaling pathways are needed in this population using targeted gene panels.

Beyond *YES/YAP1*, EMT has been shown to confer IO resistance more directly by other mechanisms. In breast cancer models, where epithelial tumor cells were infiltrated by CD8 + T cells, mesenchymal tumors contained regulatory T cells and M2 macrophages and were resistant to IO treatment with a checkpoint blockade [33]. Additionally, mixed tumors containing only a fraction of mesenchymal cells continue to recruit regulatory T cells and M2 macrophages to the primary tumor, in line with the concept of immune-cell exclusion [33, 34]. Inferred and predicted ligand-receptor pairs have been suggested, but none with cell-to-cell spatial resolution.

We demonstrated that stromal colocalization of *ITGAV* and *COL4A1* in the IO-exposed TIME is increased among fibroblasts, tumor cells, and endothelial cells. On mIF validation testing, *ITGAV* + , *SMA* + *ITGAV* + in the stroma were also seen in higher abundance following IO, suggesting a proteomic correlative increase in integrin αV among stromal fibroblasts. Further validation testing from the CPTAC dataset indicated that collagen IV α1 protein was enriched in advanced stages of disease (Fig. 7B). Methylation data from TCGA data suggests that this enrichment may be a result of DNA hypomethylation (Fig. 7D). Previously, an increase in *COL4A1* expression was noted in regions on 10 × Visium slides where metastatic colorectal cancer was colocalized with fibroblasts [35].

The *ITGAV* protein (integrin subunit αV) has a reported role in cell migration and metastasis in several cancers [36–38]. In ccRCC, integrin overexpression has been associated with tumor grade, distant metastases, and overall survival [39–43]. It has also been described in mTOR inhibitor-resistant tumors analyzed with flow cytometry [44]. The biologic role of *ITGAV* specifically in ccRCC has not yet been well characterized; however, Crona et al. identified an intronic variant of *ITGAV* that leads to overexpression and is associated with decreased OS among TKI-treated patients [43]. Feldkoren et al. used human renal-cell carcinoma cell lines to show how TGF-β-dependent overexpression of the integrin αv-β3 leads to decreased E-cadherin expression and increased cell mobility [42]. Although the biological basis of *ITGAV*’s contribution to ccRCC tumorigenesis has not yet been fully elucidated, experiments in other cancers have demonstrated an association with well described ccRCC pathways, such as VEGF-mediated neovascularization (Fig. 5C) [45–49]. Inhibition of *ITGAV* also appears to increase T-cell killing of melanoma cells in vitro [50]. Thus, the current study adds to an existing biological rationale for investigating therapeutic targets of this signaling pathway, especially among patients with ccRCC treated with IO.

Integrin targeting has been explored in other cancers [37]. Multiple agents have been developed against the integrin αv subunit, including cilengitide and abituzumab, and studied in phase II trials [37, 51, 52]. Oncologic outcomes with integrin targeting in other settings have been underwhelming thus far. In the only phase III study in glioblastoma, combination therapy with cilengitide did not improve survival over standard of care [51]. Even so, integrin-targeted therapeutics have not been tested in the IO-exposed setting in kidney cancer. This represents a potential area of combination or sequential treatment with already developed therapeutics.

The presence of extracellular matrix (ECM) components, such as collagen IV and cancer-associated fibroblasts (CAFs), have been found to play an integral role in tumor growth, migration, and neovascularization in ccRCC [53]. ECM-rich gene signatures have been shown to be associated with poorer overall survival [54]. Collagen IV specifically is a known von Hippel-Lindau interaction partner and found in abundance in ccRCC [55]. Yet the majority of ECM is produced not by cancer cells but by other stromal cell types such as fibroblasts. This fibrotic overproduction of collagen and other ECM proteins appears to be present in multiple kidney-specific pathologies, as validated in recent spot-based spatial transcriptomics [56]. In cancer, CAFs have been spatially associated with mesenchymal-like cells in ccRCC at the tumor-stromal interface on both spot-based spatial transcriptomic and proteomic analyses [57]. Thus, our findings validate this specific interaction with cellular resolution and highlight the increased autocorrelation in the IO-resistant TIME.

Our analysis also identified an increased number of dysfunctional CD8 + T cells within the stroma of the IO-exposed ccRCC TIME [58–60]. The shift of functional to dysfunctional CD8 + T-cell infiltration within the TIME over pseudotime has been well characterized [6, 58, 61–63]. The upregulation of T-cell exhaustion markers (*TOX*, *EOMES*), as well as functional T-cell responsiveness (*GZMK*) in the IO-resistant TIME in our analysis supports prior findings [61]. The mix of exhaustion and functional markers representing both exhaustion and responsiveness may reflect the unique clinical scenario of the patients from whom these samples are derived, somewhere in between complete response and secondary refractive disease.

Among sarcomatoid samples, we found a higher abundance of M2 macrophages in the stroma, which is consistent with prior studies [64]. The most significant gene expression differences in tumor FOV are found in CD8 + T cells, M2 macrophages, and regulatory T cells. Interestingly, *TGFBR2* was downregulated in tumor-embedded CD8 T cells. This gene has been shown to be associated with T-cell exhaustion in breast cancer [65]. Thus, its downregulation in sarcomatoid tumors may be one plausible mechanism for sarcomatoid tumors having favorable responses to immunotherapy. TGF-β signaling in T cells is known to regulate another significantly downregulated gene in this study, *ZFP36*, which encodes the anti-inflammatory RNA-binding protein tristetraprolin [66]. Tristetraprolin is suppressed in multiple aggressive cancers, such as breast and prostate, and can be associated with inflammatory subtypes [67]. Its dysregulation in T cells specifically is often seen in inflammatory conditions, such as rheumatoid arthritis and multiple sclerosis [68]. However, the role of tristetraprolin in cancer-associated CD8 T cells is still unclear. Additional study is required to illuminate how this specific CD8 T-cell profile could confer an exceptional response to IO therapy.

The overall role of TGF-β signaling in sarcomatoid tumors has not been conclusive [69]. Traditional GSEA of bulk RNA sequencing has not shown enrichment in the TGF-β gene set; however, Wang et al. observed upregulation of the TGF-β signaling pathway across multiple sarcomatoid samples using commercial network-based pathway analysis [64, 70]. The contrasting results of these 2 studies may be rooted in their differing methods. In the current study, we found that 3 out of 6 sarcomatoid samples were spatially enriched for TGF-β signaling, while none of the nonsarcomatoid samples were enriched. Yet in differential gene expression analysis by cell phenotype, neither TGF-β1 nor its receptor was significantly changed in expression in sarcomatoid samples. These conflicting results may represent the limitation of pathway analysis altogether or a limitation of use of partial gene sets. Although the promise of spatial transcriptomics lies in its high-plex capacity, only a portion of the hallmark gene sets could be analyzed in this study. Additional studies, with expanded gene targets, are needed to evaluate more components of the TGF-β signaling pathway and their biologic role in sarcomatoid disease.

Limitations of this study include the relatively small number of patient samples and a mixed population of treated patients, including TKI-exposed patients. Sample-size limitations, however, are bolstered by the single-cell level analysis of multiple tissue regions and by using contemporary treatment regimens. Several bioinformatics steps were developed in this study, including malignant-cell phenotyping and spatial GSEA, and require further validation in external datasets. Additionally, transcriptomic analysis was limited to genes present on the CosMx SMI platform.

## Conclusions

Using cellular-resolution spatial transcriptomics in ccRCC, we found that IO exposure is associated with increased spatial gene-set enrichment of the EMT pathway and colocalization of the ligand-receptor transcripts *COL4A1* and *ITGAV*. The cell types with the highest expression of these 2 genes were fibroblasts, tumor cells, and other endothelium cell types. Additional study is needed to elucidate the biological basis for this shift in the ccRCC TIME, as well as to examine the possible therapeutic potential of integrin following IO treatment.

## Methods

### Patient cohorts

We prospectively collected tumor samples from 21 patients with ccRCC (MCC #20,148, Advarra [Pro00038234]). The presence of sarcomatoid elements was identified on 9 patients (2 × IO exposed and 7 × IO naïve) and 12 patients without sarcomatoid features (4 × IO exposed and 8 × IO naïve). Among the 6 patients who had tumors collected after IO-based therapy, 4 patients received the combination of pembrolizumab and axitinib for a median time on therapy of 8.5 months, and 2 patients received nivolumab and ipilimumab for a median of 5.5 months. Patients exposed to IO were selected for surgical resection of their primary tumor because of concerns for remaining viable disease after initiation of IO therapy. Eight tumor samples were collected from patients without sarcomatoid elements and from those who presented with localized disease initially and underwent surgical resection (i.e., IO-naïve tumors). Clinical attributes of all patient samples can be found in Table 1. A trained pathologist (JD) identified final paired tumor and stroma (tumor-adjacent) samples: 8 × IO-naïve nonsarcomatoid, 6 × IO exposed, and 7 × IO-naïve sarcomatoid.

### TMA construction and CosMx SMI spatial gene-expression profiling

Each patient’s tumor sample had 2 spatially distinct tumor and stroma tissue samples prepared using 1-mm core biopsies for TMA creation. A total of 42 core formalin-fixed paraffin-embedded (FFPE) samples from 21 patients were allocated across 3 slides for TMA creation. We used a protocol allowing for 20 FOV at 0.9 mm × 0.7 mm that were profiled with the scanning area per slide. Full details of the CosMx SMI chemistry and workflow can be found in He et al. 2021 [71] Briefly, each slide underwent in situ hybridization (ISH) of 978 mRNA probes optimized to investigate the biology of single cells across tumors and diverse organs. Of these probes, 758 genes were selected to capture critical cell states and cell-to-cell interactions. The remaining genes and markers were selected to optimize the panel’s power to distinguish between different cell types. A list of probe targets can be found in Additional File 2: Table S16. The 4 protein markers used were CD3 (T cells), CD45 (lymphocytes), CD298 (ubiquitous human cell membrane protein), and pancytokeratin (epithelial cells) for multiplex immunofluorescence. These markers were also used for multimodal cell segmentation, which was provided by NanoString.

### Data quality control and nontumor-cell phenotyping

For each tissue sample, probe counts, spatial coordinates, and cell segmentation output was obtained and processed using open-source packages implemented in R v4.3.0 (*Seurat* v4.3.0, *InSituType* v1.0.0, and *spatialTIME* v1.3.3.3) [18, 72, 73]. Cells with fewer than 20 transcript counts or with an abnormally large cell area (resulting from segmentation errors and defined as greater than 5 times the geomentric mean) were removed from downstream analyses [74]. Additionally, FOV with fewer than 5 cells segmented were considered as failed assays and removed from data. Counts were normalized with “SCTransform” allowing removal of technical cell-to-cell variation beyond simple log-transformations [75]. Using the transformed expression data and the 4-protein fluorescence data, the *InSituType* software was used to phenotype cells based on the Kidney Cell Atlas single-cell reference [76]. Before analysis with *InSituType*, normalized gene expression of all cells in the Kidney Cell Atlas was scaled to the largest library size (i.e., cell with highest gene counts), followed by averaging of gene expression by cell phenotype.

Following *InSituType* phenotyping, T-cell and mononuclear phagocyte (MNP) clusters were subset, and identification of subclusters was performed on that subset with principal component analysis (PCA) and UMAP projections. Differentially expressed genes (DEG) among subclusters were detected with the “FindAllMarkers” function within *Seurat*. The DEG were used to further refine phenotypes (i.e., regulatory T cells, Naïve CD4 + T cells). Marker genes with positive LFC were queried in the Human Protein Atlas single-cell expression data to assign cluster identity.

### Identification of malignant cells

Given that our kidney-cell reference atlas only includes healthy cells, we could not use it for accurate identification of malignant cells. To identify malignant cells (tumor), we implemented an approach based on LASSO logistic regression of tumor marker expression. Because *VEGFA* expression has been shown to be upregulated in patients with ccRCC [77], we first selected a subset of cells from stroma and tumor FOV with distinct proximal tubule expression profiles (i.e., high or low *VEGFA* expression; Additional File 2: Table S3). Differential gene expression analysis was performed between tumor and stroma proximal tubule cells (cell of origin for ccRCC) to determine marker genes. Proximal tubule cell expression and tumor/stroma assignment based on *VEGFA* expression was passed to the function “cv.glmnet” from the R package *glmnet* (v4.1–7) to train a LASSO logistic regression model with tenfold cross-validation (L1 regularization) and providing a stronger, smaller marker gene list [78]. Using the LASSO determined marker genes, a generalized linear model (GLM) was fit and a threshold of 0.5 was used to classify a cell as tumor (< 0.5) or nontumor (≥ 0.5). This model was then applied to all cells where the phenotypes were determined from *InSituType* to be kidney tissue–related (i.e., cells determined not to be fibroblast/myofibroblast or immune-related). Additionally, on stromal FOV where the cell assignment was *glomerular endothelium* from *InSituType* and the LASSO model classified as *tumor* cell, we reassigned these cells as glomerular endothelium upon review with a genitourinary pathologist. Lastly, cell types from the Kidney Cell Atlas were collapsed to increase the number of cells in each cell type (Additional File 2: Table S17). *InSituType* phenotype assignment for glomerular endothelium was used on stromal FOV when our GLM predicted cells to be malignant follow consultation with a pathologist.

To externally validate our LASSO model’s ability to identify malignant cells with genes present in the CosMx SMI panel, we downloaded single-cell RNA sequencing data from Li et al. [17, 79]. Cell gene expression was processed the same as our CosMx data with “SCTransform” to be in the same transformed space. Genes from the LASSO model were present in the single-cell data set except for *WIF1* which may have been excluded due to zero inflation. Because of this when running the model, *WIF1* was given a value of 0 for all cells. The original authors labeled certain cell types as “RCC tumor cells,” “endothelial cells,” “proximal tubule epithelial cells,” and “nonproximal tubule epithelial cells”; these cell types were used to assess the model’s performance with area under the curve (AUC) and to assess the percent of cells correctly classified at a threshold of 0.5, the same threshold as CosMx SMI. A total of 13,105 cells were “RCC tumor cells,” and 8,954 cells belonged to the other 3 classes (total of 22,059 kidney tissue cells).

### Differential cell-type abundances

To explore differences in cell abundance between the patient groups, beta-binomial models were used to test for relationships between the number of positive cells and the treatment for primary disease, as well as IO-naïve sarcomatoid classes. *P*-values were adjusted with the Benjamini-Hochberg [80] FDR, and a threshold of 0.1 was used to identify statistical significance. To explore the finding of increase *YES1* expression in tumor cells following exposure to IO, we leveraged the public melanoma single-cell RNA-seq dataset GSE115978 [20]. Briefly, we selected author annotated malignant cells and performed log2(count + 1) transformation. Comparison between IO-naïve and IO-exposed expression was performed with a Wilcoxon rank-sum test.

### Differential univariate clustering of phenotypes

The level of spatial aggregation among cells of a given type was measured to determine differences associated with IO exposure or presence of sarcomatoid features. To do this, we implemented Ripley’s *K* count statistic form the *spatialTIME* package in R for each cell type at a radius of *r* = 150 (27 µm), and adjusting for core-specific complete spatial randomness measurement (Ripley’s *K* measured across all cells in the FOV) [81–83]. The resulting measure is the “Degree of Clustering Exact” (DOCE) and allows for comparison of values between tissue samples by removing bias introduced by areas on FOV where stationarity of the point process is violated (often due to cells not being measured in those areas). Missing measurements of cells may be due to squished tissue, FOV region expanding outside the tissue core, or necrotic tissue. The DOCE values were compared between the patient groups using a linear model predicting the DOCE from each patient group.

### Differential gene expression

We collapsed gene expression down to the FOV level by averaging all cells within a FOV (“pseudobulk”) and conducting two-sample *T* tests between sample groups [19]. Additionally, we performed differential gene expression at the cell level with “FindAllMarkers” to see if genes were differentially expressed between sample groups (e.g., *MS4A1* in B cells). To perform this analysis, a linear mixed effects model was used whereby a random-effect was used to account for cells coming from the same FOV (*lmerTest* v3.1–3) [84].

Due to the nature of our data being immunotherapy naïve and immunotherapy exposed, we identified a single-cell RNA-Seq melanoma data set with cells before and after exposure to immune checkpoint inhibitor anti-PD-1 (GSE115978) to externally validate our results. [20] Cells labeled by Jerby-Arnon et al. as Mal signify cells that are malignant and raw counts were subset from the main data for log2 transformation. Gene expression for *YES1* was compared between malignant cells before and after exposure to anti-PD-1 with Wilcoxon rank sum test.

### Spatial relationship of enrichment scores

To identify gene sets showing spatially aggregated (i.e. “hotspots”) enrichment scores in tumors from the 3 patient cohorts, we implemented a modified version of “STenrich” from the *spatialGE* R package (v1.0.0) [85]. Briefly, at the FOV level, cells with enrichment scores greater than 1 standard deviation above the mean were identified and the Euclidean distance between these identified cells was summed. The same number of identified cells was then randomly permutated 1000 times and the Euclidean distance between these permutated cell locations were summed to create an empirical null distribution. This empirical distribution representing the null hypothesis of no spatial aggregation was used to determine empirical *p*-values for the alternative hypothesis of spatial aggregation. The spatial enrichment *p*-values were then compared between patient cohorts by counting number of samples that showed significant evidence of enrichment hotspots.

### Identification of ligand-receptor spatial autocorrelation

Spatial proximity of ligand-receptor genes was evaluated using bivariate Moran’s *I*. We queried genes belonging to the EMT and IL6-JAK-STAT3 signaling pathways in the ligand-receptor pairs from *CellTalkDB*. [86] For each cell, the 3 nearest neighbors were identified with the *spdep* R package (v1.2–8) (binary weight = 1) [87]. The selection of a low number of nearest neighbors was used to ensure the association of receptor expression considered only cells in close proximity to the ligand cell. To calculate the bivariate Moran’s *I*, the ligand and receptor gene expression and weights were input to the “moran_bv” function in *spdep*. Bivariate Moran’s *I* ranges from − 1 (strong inverse spatial autocorrelation) to + 1 (strong positive spatial autocorrelation), with values around 0 indicating no spatial relationship between the gene expression values. To test for significant differences in Moran’s *I* value between the patient cohorts, 2 sample *T* tests were performed.

### Validation of ligand-receptor with multiplex immunofluorescence

To evaluate the protein expression of spatially autocorrelated transcripts, we performed multiplex immunofluorescence. Tissue samples obtained from the same TMA cores used for SMI underwent mIF using a previously described protocol [88]. In brief, FOV were stained for a panel of antibodies against PCK, SMA, ITGAV (integrin αv subunit), and COL4A1 (collagen IV), as well as DAPI nuclear counterstain. Cell positivity for specific markers was set based on previously published staining patterns and visual intensities [89]. We tested for differences in abundance of cells positive for ITGAV between patient cohorts, using non parametric Wilcoxon rank sum tests (IO naïve vs IO exposed and IO naïve vs sarcomatoid). Because of elevated expression of *COL4A1* and *ITGAV* gene expression with tumor and myofibroblasts/fibroblasts in the bivariate Moran’s *I* analysis, we looked for associations with protein expression of ITGAV on these cell types (myofibroblasts and fibroblasts identified with antibody against SMA; tumor cells identified with antibodies against PCK; Additional File 3). These associations were measured using beta-binomial models to look at positive cells between the patient cohorts with the *VGAM* R Package (v1.1–8) [90]. The COL4A1 protein is largely extracellular; hence, the total area proportion positive staining area determined visually across multiple samples was compared between IO-naïve and IO-exposed groups using Wilcoxon rank sum tests.

### CPTAC validation

Preprocessed, normalized, protein-level proteomic and phosphoproteomic data from the CPTAC clear-cell renal-cell carcinoma (ccRCC) study [21] for 110 patients was obtained from www.linkedomics.org [91] on 7/31/2023. We compared COL4A1 and ITGAV protein abundances in tumors across stages I through IV using nonparametric Wilcoxon rank sum tests.

## Pan-cancer analysis and TCGA validation

All TCGA Pan-Cancer data was all downloaded from the National Cancer Institute’s Genomic Data Commons PanCan Atlas. Tumor type and sample type were inferred from TCGA barcode. The TCGA RNA-Seq data was extracted and log2 transformed. The DNA copy-number variation–data (GISTIC data) were also extracted. Using the GISTIC2.0 DNA copy-number analysis, the following numeric values were assigned with the cBioPortal interpretation presented in parenthesis: − 2 = Deep Deletion (possibly a homozygous deletion); − 1 = Shallow Deletion (possible a heterozygous deletion); 0 = Diploid; + 1 = Gain (a few additional copies, often broad); + 2 = Amplification (more copies, often focal). For data present in this chapter, only samples with Illumina’s Infinium HumanMethylation450 BeadChip data were used. Raw IDAT files were downloaded from TCGA. Preprocessing the data included normalization via internal controls followed by background subtraction using the methylumi R package.

For a full list of resources, please see Key Resources table in Additional File 3.

## Supplementary Information

Additional File 1. Figs. S1, S2, S3. Fig S1. Study overview showing the number of samples per group, data generation with CosMx Spatial Molecular Imaging, cell-type identification, and analyses. Fig S2. Abundance of final cell assignments. (A) shows IO naïve primary nonsarcomatoid; (B) shows IO exposed; and (C) shows IO naïve primary sarcomatoid stroma and tumor FOV. Fig S3. Expression of *COL4A1* and *ITGAV* in normal tissues from GTEx project. Abbreviation: GTEx, Genotype-Tissue Expression.

Additional File 2. Tables S1, S2, S3, S4, S5, S6, S7, S8, S9, S10, S11, S12, S13, S14, S15, S16, S17: Table S1. Number of Cells That Passed Quality Control on Primary Tumor FOV. Abbreviation: FOV, fields of view; Table S2. Differentially Expressed Genes Identified From Subclustering InSituType-Assigned T Cells and Mononucleic Phagocytes; Table S3. Differential Gene Expression Using FindMarkers Between Malignant Proximal Tubule and Normal Proximal Tubule Cells. These cells were used for LASSO regression and genes selected by LASSO for final model. Abbreviation: LASSO, least absolute shrinkage and selection operator; Table S4. Abundance Differences for Cell Phenotypes in the Tumor and Stromal FOV of the Cohorts. Positive estimate indicates lower abundance in IO-naïve nonsarcomatoid. Abbreviations: FOV, fields of view; IO, immunotherapy; Table S5. Association of Ripley’s K Between Cohorts in Tumor and Stromal FOV Tissue at a Radius of 150 px (27 µm). Negative-estimate values indicate lower clustering in IO-naïve nonsarcomatoid samples. Abbreviations: FOV, fields of view; IO, immunotherapy; Table S6. Differential Gene Expression of Primary ccRCC Tumor and Stroma Samples Between Cohorts. Abbreviation: ccRCC, clear-cell renal-cell carcinoma; Table S7. Differential Gene Expression for Each Cell Phenotype in Primary-Tumor FOV Between Pre- and Post-IO. Abbreviations: FOV, fields of view; IO, immunotherapy; Table S8. Differential Gene Expression for Each Phenotype on Primary Tumor FOV Before Undergoing IO With and Without Sarcomatoid Features. Abbreviations: FOV, fields of view; IO, immunotherapy; Table S9. Differential Gene Expression in Stromal FOV, Between Pre- and Post-IO Primary Samples. Abbreviations: FOV, fields of view; IO, immunotherapy; Table S10. Differential Gene Expression for Each Phenotype on Primary Stromal FOV Before Undergoing IO With and Without Sarcomatoid Features. Abbreviations: FOV, fields of view; IO, immunotherapy; Table S2. Results of the Spatial Enrichment of Hallmark Gene Sets in All Samples; Table S12. T-Test Results Comparing Bivariate Moran’s I of Ligand/Receptor Pairs From EMT Gene Set in Tumor and Stromal FOV. Abbreviations: EMT, epithelial-mesenchymal transition; FOV, fields of view; Table S13. T-Test Results Comparing Bivariate Moran’s I of Ligand/Receptor Pairs From IL6/JAK/STAT3 Signaling Gene-Set Tumor and Stromal FOV. Abbreviation: FOV, fields of view; Table S14. Comparisons Between ITGAV and COL4A1 Abundance in the Tumor and Stromal Compartments; Table S15. Results of Beta-Binomial Modeling of Phenotypes Between Cohorts of the Validation mIF. Abbreviation: mIF, multiplex immunofluorescence; Table S16. Nanostrisng CosMx SMI Probe Targets; Table S17. Kidney Cell Atlas Phenotype Names Before (InsituType_Manual) and After (Final) Collapsing Similar Cell Types.

Additional File 3. Information related to antibodies and stains used for multiplex immunofluorescence, reagents and equipment used for the imaging and Nanostring Spatial Molecular Imager platform, location of deposited data and code, and R packages/versions used for analysis.

Additional File 4. Review history.

## Data Availability

Data produced by Nanostring CosMx SMI platform is available on Zenodo at https://zenodo.org/records/12730227 [92], the multiplex data is available in the form of a *spatialTIME* mIF object on Zenodo at https://zenodo.org/records/13890928 [93], and code used to process, annotate, and analyze the data can be found on GitHub (https://github.com/FridleyLab/ccRCC_CosMx_SMI_2023) or Zenodo at https://zenodo.org/records/13891052 under a CC BY 4.0 license [94]. For relevant clinical or additional information required to reanalyze the data reported is available from BJM upon request.

## References

[1] Motzer RJ, Tannir NM, McDermott DF, Arén Frontera O, Melichar B, Choueiri TK, Plimack ER, Barthélémy P, Porta C, George S, et al. Nivolumab plus ipilimumab versus sunitinib in advanced renal-cell carcinoma. N Engl J Med. 2018;378:1277–90. 29562145 10.1056/NEJMoa1712126 PMC5972549

[2] Rini BI, Plimack ER, Stus V, Gafanov R, Hawkins R, Nosov D, Pouliot F, Alekseev B, Soulières D, Melichar B, et al. Pembrolizumab plus axitinib versus sunitinib for advanced renal-cell carcinoma. N Engl J Med. 2019;380:1116–27. 30779529 10.1056/NEJMoa1816714

[3] Albiges L, Tannir NM, Burotto M, McDermott D, Plimack ER, Barthélémy P, Porta C, Powles T, Donskov F, George S, et al. Nivolumab plus ipilimumab versus sunitinib for first-line treatment of advanced renal cell carcinoma: extended 4-year follow-up of the phase III CheckMate 214 trial. ESMO Open. 2020;5: e001079. 33246931 10.1136/esmoopen-2020-001079 PMC7703447

[4] Tannir NM, Signoretti S, Choueiri TK, McDermott DF, Motzer RJ, Flaifel A, Pignon JC, Ficial M, Frontera OA, George S, et al. Efficacy and safety of nivolumab plus ipilimumab versus sunitinib in first-line treatment of patients with advanced sarcomatoid renal cell carcinoma. Clin Cancer Res. 2021;27:78–86. 32873572 10.1158/1078-0432.CCR-20-2063 PMC8589223

[5] Miao D, Margolis CA, Gao W, Voss MH, Li W, Martini DJ, Norton C, Bossé D, Wankowicz SM, Cullen D, et al. Genomic correlates of response to immune checkpoint therapies in clear cell renal cell carcinoma. Science. 2018;359:801–6. 29301960 10.1126/science.aan5951 PMC6035749

[6] Chakiryan NH, Kim Y, Berglund A, Chang A, Kimmel GJ, Hajiran A, Nguyen J, Moran-Segura C, Saeed-Vafa D, Katende EN, et al. Geospatial characterization of immune cell distributions and dynamics across the microenvironment in clear cell renal cell carcinoma. J Immunother Cancer. 2023;11: e006195. 37185232 10.1136/jitc-2022-006195 PMC10151991

[7] Hakimi AA, Voss MH, Kuo F, Sanchez A, Liu M, Nixon BG, Vuong L, Ostrovnaya I, Chen Y-B, Reuter V, et al. Transcriptomic profiling of the tumor microenvironment reveals distinct subgroups of clear cell renal cell cancer: data from a randomized phase III trial. Cancer Discov. 2019;9:510–25. 30622105 10.1158/2159-8290.CD-18-0957 PMC6697163

[8] Chevrier S, Levine JH, Zanotelli VRT, Silina K, Schulz D, Bacac M, Ries CH, Ailles L, Jewett MAS, Moch H, et al. An immune atlas of clear cell renal cell carcinoma. Cell. 2017;169:736-749.e718. 28475899 10.1016/j.cell.2017.04.016 PMC5422211

[9] Şenbabaoğlu Y, Gejman RS, Winer AG, Liu M, Van Allen EM, de Velasco G, Miao D, Ostrovnaya I, Drill E, Luna A, et al. Tumor immune microenvironment characterization in clear cell renal cell carcinoma identifies prognostic and immunotherapeutically relevant messenger RNA signatures. Genome Biol. 2016;17:231. 27855702 10.1186/s13059-016-1092-z PMC5114739

[10] Young MD, Mitchell TJ, Vieira Braga FA, Tran MGB, Stewart BJ, Ferdinand JR, Collord G, Botting RA, Popescu DM, Loudon KW, et al. Single-cell transcriptomes from human kidneys reveal the cellular identity of renal tumors. Science. 2018;361:594–9. 30093597 10.1126/science.aat1699 PMC6104812

[11] Krishna C, DiNatale RG, Kuo F, Srivastava RM, Vuong L, Chowell D, Gupta S, Vanderbilt C, Purohit TA, Liu M, et al. Single-cell sequencing links multiregional immune landscapes and tissue-resident T cells in ccRCC to tumor topology and therapy efficacy. Cancer Cell. 2021;39:662-677.e666. 33861994 10.1016/j.ccell.2021.03.007 PMC8268947

[12] Zhang Y, Narayanan SP, Mannan R, Raskind G, Wang X, Vats P, Su F, Hosseini N, Cao X, Kumar-Sinha C, et al. Single-cell analyses of renal cell cancers reveal insights into tumor microenvironment, cell of origin, and therapy response. Proc Natl Acad Sci U S A. 2021;118:e2103240118. 34099557 10.1073/pnas.2103240118 PMC8214680

[13] Liu J, Tran V, Vemuri VNP, Byrne A, Borja M, Kim YJ, Agarwal S, Wang R, Awayan K, Murti A, et al. Concordance of MERFISH spatial transcriptomics with bulk and single-cell RNA sequencing. Life Sci Alliance. 2023;6. 10.26508/lsa.202201701. 10.26508/lsa.202201701 PMC9760489 36526371

[14] He S, Bhatt R, Brown C, Brown EA, Buhr DL, Chantranuvatana K, Danaher P, Dunaway D, Garrison RG, Geiss G, et al. High-plex imaging of RNA and proteins at subcellular resolution in fixed tissue by spatial molecular imaging. Nat Biotechnol. 2022;40:1794–806. 36203011 10.1038/s41587-022-01483-z

[15] Chahoud J, Anderson ARA, Zhang J, Brown J, Gatenby RA. Evolutionary dynamics and intermittent therapy for metastatic cancers. J Clin Oncol. 2023;41:4469–71. 37418680 10.1200/JCO.23.00647 PMC10553063

[16] Gatenby RA, Brown JS. The evolution and ecology of resistance in cancer therapy. Cold Spring Harb Perspect Med. 2020;10. 10.1101/cshperspect.a040972. 10.1101/cshperspect.a040972 PMC7605238 33139405

[17] Li R, Ferdinand JR, Loudon KW, Bowyer GS, Laidlaw S, Muyas F, Mamanova L, Neves JB, Bolt L, Fasouli ES, et al. Mapping single-cell transcriptomes in the intra-tumoral and associated territories of kidney cancer. Cancer Cell. 2022;40:1583-1599.e1510. 36423636 10.1016/j.ccell.2022.11.001 PMC9767677

[18] Creed JH, Wilson CM, Soupir AC, Colin-Leitzinger CM, Kimmel GJ, Ospina OE, Chakiryan NH, Markowitz J, Peres LC, Coghill A, Fridley BL. spatialTIME and iTIME: R package and Shiny application for visualization and analysis of immunofluorescence data. Bioinformatics. 2021;37:4584–6. 34734969 10.1093/bioinformatics/btab757 PMC8652029

[19] Lähnemann D, Köster J, Szczurek E, McCarthy DJ, Hicks SC, Robinson MD, Vallejos CA, Campbell KR, Beerenwinkel N, Mahfouz A, et al. Eleven grand challenges in single-cell data science. Genome Biol. 2020;21:31. 32033589 10.1186/s13059-020-1926-6 PMC7007675

[20] Jerby-Arnon L, Shah P, Cuoco MS, Rodman C, Su MJ, Melms JC, Leeson R, Kanodia A, Mei S, Lin JR, et al. A cancer cell program promotes T cell exclusion and resistance to checkpoint blockade. Cell. 2018;175:984-997.e924. 30388455 10.1016/j.cell.2018.09.006 PMC6410377

[21] Clark DJ, Dhanasekaran SM, Petralia F, Pan J, Song X, Hu Y, da Veiga LF, Reva B, Lih TM, Chang HY, et al. Integrated proteogenomic characterization of clear cell renal cell carcinoma. Cell. 2019;179:964-983.e931. 31675502 10.1016/j.cell.2019.10.007 PMC7331093

[22] Lonsdale J, Thomas J, Salvatore M, Phillips R, Lo E, Shad S, Hasz R, Walters G, Garcia F, Young N, et al. The Genotype-Tissue Expression (GTEx) project. Nat Genet. 2013;45:580–5. 23715323 10.1038/ng.2653 PMC4010069

[23] Nallandhighal S, Vince R, Karim R, Groves S, Stangl-Kremser J, Russell C, Hu K, Pham T, Cani AK, Liu CJ, et al. Molecular characterization of clear cell renal cell carcinoma reveals prognostic significance of epithelial-mesenchymal transition gene expression signature. Eur Urol Oncol. 2022;5:92–9. 34840106 10.1016/j.euo.2021.10.007

[24] Zhang Y, Narayanan SP, Mannan R, Raskind G, Wang X, Vats P, Su F, Hosseini N, Cao X, Kumar-Sinha C, et al. Single-cell analyses of renal cell cancers reveal insights into tumor microenvironment, cell of origin, and therapy response. Proc Natl Acad Sci U SA. 2021;118:e2103240118. 10.1073/pnas.2103240118 PMC8214680 34099557

[25] Liu F, Wang P, Sun W, Jiang Y, Gong Q. Identification of ligand-receptor pairs associated with tumour characteristics in clear cell renal cell carcinoma. Front Immunol. 2022;13:874056. 35734169 10.3389/fimmu.2022.874056 PMC9207243

[26] Wu Y, Terekhanova NV, Caravan W, Naser Al Deen N, Lal P, Chen S, Mo CK, Cao S, Li Y, Karpova A, et al. Epigenetic and transcriptomic characterization reveals progression markers and essential pathways in clear cell renal cell carcinoma. Nat Commun. 2023;14:1681. 36973268 10.1038/s41467-023-37211-7 PMC10042888

[27] Kalluri R. The biology and function of fibroblasts in cancer. Nat Rev Cancer. 2016;16:582–98. 27550820 10.1038/nrc.2016.73

[28] Rybarczyk A, Klacz J, Wronska A, Matuszewski M, Kmiec Z, Wierzbicki PM. Overexpression of the YAP1 oncogene in clear cell renal cell carcinoma is associated with poor outcome. Oncol Rep. 2017;38:427–39. 28504812 10.3892/or.2017.5642

[29] Poma AM, Torregrossa L, Bruno R, Basolo F, Fontanini G. Hippo pathway affects survival of cancer patients: extensive analysis of TCGA data and review of literature. Sci Rep. 2018;8:10623. 30006603 10.1038/s41598-018-28928-3 PMC6045671

[30] Chen X, Zhang X, Jiang Y, Zhang X, Liu M, Wang S, Liu S, Liang H, Liu C. YAP1 activation promotes epithelial-mesenchymal transition and cell survival of renal cell carcinoma cells under shear stress. Carcinogenesis. 2022;43:301–10. 35147702 10.1093/carcin/bgac014

[31] Nguyen CDK, Yi C. YAP/TAZ signaling and resistance to cancer therapy. Trends Cancer. 2019;5:283–96. 31174841 10.1016/j.trecan.2019.02.010 PMC6557283

[32] Tucker MD, Rini BI. Predicting response to immunotherapy in metastatic renal cell carcinoma. Cancers (Basel). 2020;12:12. 10.3390/cancers12092662 PMC7565517 32961934

[33] Dongre A, Rashidian M, Reinhardt F, Bagnato A, Keckesova Z, Ploegh HL, Weinberg RA. Epithelial-to-mesenchymal transition contributes to immunosuppression in breast carcinomas. Cancer Res. 2017;77:3982–9. 28428275 10.1158/0008-5472.CAN-16-3292 PMC5541771

[34] Joyce JA, Fearon DT. T cell exclusion, immune privilege, and the tumor microenvironment. Science. 2015;348:74–80. 25838376 10.1126/science.aaa6204

[35] Mason K, Sathe A, Hess PR, Rong J, Wu C-Y, Furth E, Susztak K, Levinsohn J, Ji HP, Zhang N. Niche-DE: niche-differential gene expression analysis in spatial transcriptomics data identifies context-dependent cell-cell interactions. Genome Biol. 2024;25:14. 38217002 10.1186/s13059-023-03159-6 PMC10785550

[36] Waisberg J, De Souza VL, Affonso Junior RJ, Silva SR, Denadai MV, Margeotto FB, De Souza CS, Matos D. Overexpression of the ITGAV gene is associated with progression and spread of colorectal cancer. Anticancer Res. 2014;34:5599–607. 25275062

[37] Cheuk IW, Siu MT, Ho JC, Chen J, Shin VY, Kwong A. ITGAV targeting as a therapeutic approach for treatment of metastatic breast cancer. Am J Cancer Res. 2020;10:211–23. 32064162 PMC7017729

[38] Loeser H, Scholz M, Fuchs H, Essakly A, Damanakis AI, Zander T, Büttner R, Schröder W, Bruns C, Quaas A, Gebauer F. Integrin alpha V (ITGAV) expression in esophageal adenocarcinoma is associated with shortened overall-survival. Sci Rep. 2020;10:18411. 33110104 10.1038/s41598-020-75085-7 PMC7591891

[39] Breuksch I, Prosinger F, Baehr F, Engelhardt FP, Bauer HK, Thüroff JW, Heimes AS, Hasenburg A, Prawitt D, Brenner W. Integrin α5 triggers the metastatic potential in renal cell carcinoma. Oncotarget. 2017;8:107530–42. 29296184 10.18632/oncotarget.22501 PMC5746086

[40] Haber T, Jöckel E, Roos FC, Junker K, Prawitt D, Hampel C, Thüroff JW, Brenner W. Bone metastasis in renal cell carcinoma is preprogrammed in the primary tumor and caused by AKT and integrin α5 signaling. J Urol. 2015;194:539–46. 25623744 10.1016/j.juro.2015.01.079

[41] Chen Y, Wang Y, Cai Z, Fan X, Zhang Y. Integrin α7 is overexpressed and correlates with higher pathological grade, increased T stage, advanced TNM stage as well as worse survival in clear cell renal cell carcinoma patients: a retrospective study. J Clin Lab Anal. 2020;34:e23034. 31713264 10.1002/jcla.23034 PMC6977402

[42] Feldkoren B, Hutchinson R, Rapoport Y, Mahajan A, Margulis V. Integrin signaling potentiates transforming growth factor-beta 1 (TGF-β1) dependent down-regulation of E-Cadherin expression - Important implications for epithelial to mesenchymal transition (EMT) in renal cell carcinoma. Exp Cell Res. 2017;355:57–66. 28363829 10.1016/j.yexcr.2017.03.051

[43] Crona DJ, Skol AD, Leppänen VM, Glubb DM, Etheridge AS, Hilliard E, Peña CE, Peterson YK, Klauber-DeMore N, Alitalo KK, Innocenti F. Genetic variants of VEGFA and FLT4 are determinants of survival in renal cell carcinoma patients treated with sorafenib. Cancer Res. 2019;79:231–41. 30385613 10.1158/0008-5472.CAN-18-1089 PMC6541205

[44] Juengel E, Makarević J, Reiter M, Mani J, Tsaur I, Bartsch G, Haferkamp A, Blaheta RA. Resistance to the mTOR inhibitor temsirolimus alters adhesion and migration behavior of renal cell carcinoma cells through an integrin α5- and integrin β3-dependent mechanism. Neoplasia. 2014;16:291–300. 24862756 10.1016/j.neo.2014.03.011 PMC4094828

[45] Lima ESR, Mirando AC, Tzeng SY, Green JJ, Popel AS, Pandey NB, Campochiaro PA. Anti-angiogenic collagen IV-derived peptide target engagement with α(v)β(3) and α(5)β(1) in ocular neovascularization models. iScience. 2023;26:106078. 36844452 10.1016/j.isci.2023.106078 PMC9947312

[46] Brooks PC, Clark RA, Cheresh DA. Requirement of vascular integrin alpha v beta 3 for angiogenesis. Science. 1994;264:569–71. 7512751 10.1126/science.7512751

[47] Huang R, Rofstad EK. Integrins as therapeutic targets in the organ-specific metastasis of human malignant melanoma. J Exp Clin Cancer Res. 2018;37:92. 29703238 10.1186/s13046-018-0763-x PMC5924434

[48] Pedchenko V, Zent R, Hudson BG. Alpha(v)beta3 and alpha(v)beta5 integrins bind both the proximal RGD site and non-RGD motifs within noncollagenous (NC1) domain of the alpha3 chain of type IV collagen: implication for the mechanism of endothelia cell adhesion. J Biol Chem. 2004;279:2772–80. 14610079 10.1074/jbc.M311901200

[49] Kumar CC, Malkowski M, Yin Z, Tanghetti E, Yaremko B, Nechuta T, Varner J, Liu M, Smith EM, Neustadt B, et al. Inhibition of angiogenesis and tumor growth by SCH221153, a dual alpha(v)beta3 and alpha(v)beta5 integrin receptor antagonist. Cancer Res. 2001;61:2232–8. 11280792

[50] Kishton RJ, Patel SJ, Decker AE, Vodnala SK, Cam M, Yamamoto TN, Patel Y, Sukumar M, Yu Z, Ji M, et al. Cancer genes disfavoring T cell immunity identified via integrated systems approach. Cell Rep. 2022;40: 111153. 35926468 10.1016/j.celrep.2022.111153 PMC9402397

[51] Stupp R, Hegi ME, Gorlia T, Erridge SC, Perry J, Hong YK, Aldape KD, Lhermitte B, Pietsch T, Grujicic D, et al. Cilengitide combined with standard treatment for patients with newly diagnosed glioblastoma with methylated MGMT promoter (CENTRIC EORTC 26071–22072 study): a multicentre, randomised, open-label, phase 3 trial. Lancet Oncol. 2014;15:1100–8. 25163906 10.1016/S1470-2045(14)70379-1

[52] Jiang Y, Dai J, Yao Z, Shelley G, Keller ET. Abituzumab targeting of αV-class integrins inhibits prostate cancer progression. Mol Cancer Res. 2017;15:875–83. 28314844 10.1158/1541-7786.MCR-16-0447 PMC5541673

[53] Chakiryan NH, Kimmel GJ, Kim Y, Johnson JO, Clark N, Hajiran A, Chang A, Aydin AM, Zemp L, Katende E, et al. Geospatial cellular distribution of cancer-associated fibroblasts significantly impacts clinical outcomes in metastatic clear cell renal cell carcinoma. Cancers (Basel). 2021;13:3743. 34359645 10.3390/cancers13153743 PMC8345222

[54] Ahluwalia P, Ahluwalia M, Mondal AK, Sahajpal N, Kota V, Rojiani MV, Rojiani AM, Kolhe R. Prognostic and therapeutic implications of extracellular matrix associated gene signature in renal clear cell carcinoma. Sci Rep. 2021;11:7561. 33828127 10.1038/s41598-021-86888-7 PMC8026590

[55] Oxburgh L. The extracellular matrix environment of clear cell renal cell carcinoma. Cancers (Basel). 2022;14:14. 10.3390/cancers14174072 PMC9454539 36077607

[56] Lake BB, Menon R, Winfree S, Hu Q, Melo Ferreira R, Kalhor K, Barwinska D, Otto EA, Ferkowicz M, Diep D, et al. An atlas of healthy and injured cell states and niches in the human kidney. Nature. 2023;619:585–94. 37468583 10.1038/s41586-023-05769-3 PMC10356613

[57] Davidson G, Helleux A, Vano YA, Lindner V, Fattori A, Cerciat M, Elaidi RT, Verkarre V, Sun CM, Chevreau C, et al. Mesenchymal-like tumor cells and myofibroblastic cancer-associated fibroblasts are associated with progression and immunotherapy response of clear-cell renal cell carcinoma. Cancer Res. 2023. 10.1158/0008-5472.CAN-22-3034 37335139

[58] Braun DA, Street K, Burke KP, Cookmeyer DL, Denize T, Pedersen CB, Gohil SH, Schindler N, Pomerance L, Hirsch L, et al. Progressive immune dysfunction with advancing disease stage in renal cell carcinoma. Cancer Cell. 2021;39:632-648.e638. 10.1158/0008-5472.CAN-22-3034. 10.1016/j.ccell.2021.02.013 PMC8138872 33711273

[59] Scott AC, Dündar F, Zumbo P, Chandran SS, Klebanoff CA, Shakiba M, Trivedi P, Menocal L, Appleby H, Camara S, et al. TOX is a critical regulator of tumour-specific T cell differentiation. Nature. 2019;571:270–4. 31207604 10.1038/s41586-019-1324-y PMC7698992

[60] Bi K, He MX, Bakouny Z, Kanodia A, Napolitano S, Wu J, Grimaldi G, Braun DA, Cuoco MS, Mayorga A, et al. Tumor and immune reprogramming during immunotherapy in advanced renal cell carcinoma. Cancer Cell. 2021;39:649-661.e645. 33711272 10.1016/j.ccell.2021.02.015 PMC8115394

[61] Au L, Hatipoglu E, Robert de Massy M, Litchfield K, Beattie G, Rowan A, Schnidrig D, Thompson R, Byrne F, Horswell S, et al. Determinants of anti-PD-1 response and resistance in clear cell renal cell carcinoma. Cancer Cell. 2021;39:1497-1518.e1411. 34715028 10.1016/j.ccell.2021.10.001 PMC8599450

[62] Miller BC, Sen DR, Al Abosy R, Bi K, Virkud YV, LaFleur MW, Yates KB, Lako A, Felt K, Naik GS, et al. Subsets of exhausted CD8(+) T cells differentially mediate tumor control and respond to checkpoint blockade. Nat Immunol. 2019;20:326–36. 30778252 10.1038/s41590-019-0312-6 PMC6673650

[63] Braun DA, Hou Y, Bakouny Z, Ficial M, Sant’ Angelo M, Forman J, Ross-Macdonald P, Berger AC, Jegede OA, Elagina L, et al. Interplay of somatic alterations and immune infiltration modulates response to PD-1 blockade in advanced clear cell renal cell carcinoma. Nat Med. 2020;26:909–18. 32472114 10.1038/s41591-020-0839-y PMC7499153

[64] Bakouny Z, Braun DA, Shukla SA, Pan W, Gao X, Hou Y, Flaifel A, Tang S, Bosma-Moody A, He MX, et al. Integrative molecular characterization of sarcomatoid and rhabdoid renal cell carcinoma. Nat Commun. 2021;12:808. 33547292 10.1038/s41467-021-21068-9 PMC7865061

[65] Xie F, Zhou X, Su P, Li H, Tu Y, Du J, Pan C, Wei X, Zheng M, Jin K, et al. Breast cancer cell-derived extracellular vesicles promote CD8(+) T cell exhaustion via TGF-β type II receptor signaling. Nat Commun. 2022;13:4461. 35915084 10.1038/s41467-022-31250-2 PMC9343611

[66] Ogawa K, Chen F, Kim YJ, Chen Y. Transcriptional regulation of tristetraprolin by transforming growth factor-beta in human T cells. J Biol Chem. 2003;278:30373–81. 12754205 10.1074/jbc.M304856200

[67] Brennan SE, Kuwano Y, Alkharouf N, Blackshear PJ, Gorospe M, Wilson GM. The mRNA-destabilizing protein tristetraprolin is suppressed in many cancers, altering tumorigenic phenotypes and patient prognosis. Cancer Res. 2009;69:5168–76. 19491267 10.1158/0008-5472.CAN-08-4238 PMC2724875

[68] Makita S, Takatori H, Nakajima H. Post-transcriptional regulation of immune responses and inflammatory diseases by RNA-binding ZFP36 family proteins. Front Immunol. 2021;12:711633. 34276705 10.3389/fimmu.2021.711633 PMC8282349

[69] Blum KA, Gupta S, Tickoo SK, Chan TA, Russo P, Motzer RJ, Karam JA, Hakimi AA. Sarcomatoid renal cell carcinoma: biology, natural history and management. Nat Rev Urol. 2020;17:659–78. 33051619 10.1038/s41585-020-00382-9 PMC7551522

[70] Wang Z, Kim TB, Peng B, Karam J, Creighton C, Joon A, Kawakami F, Trevisan P, Jonasch E, Chow CW, et al. Sarcomatoid renal cell carcinoma has a distinct molecular pathogenesis, driver mutation profile, and transcriptional landscape. Clin Cancer Res. 2017;23:6686–96. 28710314 10.1158/1078-0432.CCR-17-1057 PMC5683086

[71] Shanshan H, Ruchir B, Carl B, Emily AB, Derek LB, Kan C, Patrick D, Dwayne D, Ryan GG, Gary G, et al. High-plex multiomic analysis in FFPE at subcellular level by spatial molecular imaging. bioRxiv. 2022:2021.2011.2003.467020. https://www.biorxiv.org/content/10.1101/2021.11.03.467020v3.

[72] Hao Y, Hao S, Andersen-Nissen E, Mauck WM, Zheng S, Butler A, Lee MJ, Wilk AJ, Darby C, Zager M, et al. Integrated analysis of multimodal single-cell data. Cell. 2021;184:3573-3587.e3529. 34062119 10.1016/j.cell.2021.04.048 PMC8238499

[73] Danaher P, Zhao E, Yang Z, Ross D, Gregory M, Reitz Z, Kim TK, Baxter S, Jackson S, He S, et al. Insitutype: likelihood-based cell typing for single cell spatial transcriptomics. bioRxiv. 2022.10.19.512902. 10.1101/2022.10.19.512902.

[74] Garrido-Trigo A, Corraliza AM, Veny M, Dotti I, Melón-Ardanaz E, Rill A, Crowell HL, Corbí Á, Gudiño V, Esteller M, et al. Macrophage and neutrophil heterogeneity at single-cell spatial resolution in human inflammatory bowel disease. Nat Commun. 2023;14:4506. 37495570 10.1038/s41467-023-40156-6 PMC10372067

[75] Hafemeister C, Satija R. Normalization and variance stabilization of single-cell RNA-seq data using regularized negative binomial regression. Genome Biol. 2019;20:296. 31870423 10.1186/s13059-019-1874-1 PMC6927181

[76] Stewart BJ, Ferdinand JR, Young MD, Mitchell TJ, Loudon KW, Riding AM, Richoz N, Frazer GL, Staniforth JUL, Vieira Braga FA, et al. Spatiotemporal immune zonation of the human kidney. Science. 2019;365:1461–6. 31604275 10.1126/science.aat5031 PMC7343525

[77] Situ Y, Xu Q, Deng L, Zhu Y, Gao R, Lei L, Shao Z. System analysis of VEGFA in renal cell carcinoma: the expression, prognosis, gene regulation network and regulation targets. Int J Biol Markers. 2022;37:90–101. 34870494 10.1177/17246008211063501

[78] Tay JK, Narasimhan B, Hastie T. Elastic net regularization paths for all generalized linear models. J Stat Softw. 2023;106:1–31. 37138589 10.18637/jss.v106.i01 PMC10153598

[79] Mitchell TJL, Ruoyan. Mapping single cell trascriptomics in kidney cancer. Mendeley data. 2022. 10.17632/g67bkbnhhg.1.

[80] Storey JD. A direct approach to false discovery rates. J R Stat Soc Ser B Stat Methodol. 2002;64:479–98.

[81] Kiskowski MA, Hancock JF, Kenworthy AK. On the use of Ripley’s K-function and its derivatives to analyze domain size. Biophys J. 2009;97:1095–103. 19686657 10.1016/j.bpj.2009.05.039 PMC2726315

[82] Wilson C, Soupir AC, Thapa R, Creed J, Nguyen J, Segura CM, Gerke T, Schildkraut JM, Peres LC, Fridley BL. Tumor immune cell clustering and its association with survival in African American women with ovarian cancer. PLoS Comput Biol. 2022;18: e1009900. 35235563 10.1371/journal.pcbi.1009900 PMC8920290

[83] Ripley BD. Spatial statistics: developments 1980–3, correspondent paper. International Statistical Review / Revue Internationale de Statistique. 1984;52:141–50.

[84] Kuznetsova A, Brockhoff PB, Christensen RHB. lmerTest package: tests in linear mixed effects models. J Stat Softw. 2017;82:1–26.

[85] Ospina OE, Wilson CM, Soupir AC, Berglund A, Smalley I, Tsai KY, Fridley BL. spatialGE: quantification and visualization of the tumor microenvironment heterogeneity using spatial transcriptomics. Bioinformatics. 2022;38:2645–7. 35258565 10.1093/bioinformatics/btac145 PMC9890305

[86] Shao X, Liao J, Li C, Lu X, Cheng J, Fan X. CellTalkDB: a manually curated database of ligand–receptor interactions in humans and mice. Brief Bioinform. 2020;22:bbaa269. 10.1093/bib/bbaa269 33147626

[87] Bivand R. R packages for analyzing spatial data: a comparative case study with areal data. Geogr Anal. 2022;54:488–518.

[88] Nicholas HC, Youngchul K, Anders B, Andrew C, Gregory JK, Ali H, Jonathan N, Carlos MS, Daryoush SV, Esther NK, et al. Geospatial characterization of immune cell distributions and dynamics across the microenvironment in clear cell renal cell carcinoma. J Immunother Cancer. 2023;11:e006195. 37185232 10.1136/jitc-2022-006195 PMC10151991

[89] Parra ER, Uraoka N, Jiang M, Cook P, Gibbons D, Forget M-A, Bernatchez C, Haymaker C, Wistuba II, Rodriguez-Canales J. Validation of multiplex immunofluorescence panels using multispectral microscopy for immune-profiling of formalin-fixed and paraffin-embedded human tumor tissues. Sci Rep. 2017;7:13380. 29042640 10.1038/s41598-017-13942-8 PMC5645415

[90] Yee TW. The VGAM package for categorical data analysis. J Stat Softw. 2010;32:1–34.

[91] Vasaikar SV, Straub P, Wang J, Zhang B. LinkedOmics: analyzing multi-omics data within and across 32 cancer types. Nucleic Acids Res. 2018;46:D956-d963. 29136207 10.1093/nar/gkx1090 PMC5753188

[92] Soupir A, Hayes M, Peak T, Ospina O, Chakiryan N, Berglund A, Stewart P, Nguyen J, Segura CM, Francis N, et al. Increased spatial coupling of integrin and collagen IV in the immunoresistant clear-cell renal-cell carcinoma tumor microenvironment - nanostring CosMx SMI Data. Zenodo. 2024. 10.5281/zenodo.12730227. 10.1186/s13059-024-03435-z PMC11622564 39639369

[93] Soupir A, Hayes M, Peak T, Ospina O, Chakiryan N, Berglund A, Stewart P, Nguyen J, Segura CM, Francis N, et al. Increased spatial coupling of integrin and collagen IV in the immunoresistant clear-cell renal-cell carcinoma tumor microenvironment - validation mIF. Zenodo. 2024. 10.5281/zenodo.13890928. 10.1186/s13059-024-03435-z PMC11622564 39639369

[94] Soupir A, Hayes M, Peak T, Ospina O, Chakiryan N, Berglund A, Stewart P, Nguyen J, Segura CM, Francis N, et al. Increased spatial coupling of integrin and collagen IV in the immunoresistant clear-cell renal-cell carcinoma tumor microenvironment - R code. Zenodo. 2024. 10.5281/zenodo.13891052. 10.1186/s13059-024-03435-z PMC11622564 39639369

