# Supplementary material for: Increased spatial coupling of integrin and collagen IV in the immunoresistant clear-cell renal-cell carcinoma tumor microenvironment
Source: Genome Biol. 2024 Dec 5;25:308. doi: 10.1186/s13059-024-03435-z (PMC11622564; doi:10.1186/s13059-024-03435-z)
Supplement: Supplementary file 1 — Additional File 1. Figs. S1, S2, S3. Fig S1. Study overview showing the number of samples per group, data generation with CosMx Spatial Molecular Imaging, cell-type identification, and analyses. Fig S2. Abundance of final cell assignments. (A) shows IO naïve primary nonsarcomatoid; (B) shows IO exposed; and (C) shows IO naïve primary sarcomatoid stroma and tumor FOV. Fig S3. Expression of COL4A1 and ITGAV in normal tissues from GTEx project. Abbreviation: GTEx, Genotype-Tissue Expression. [file 13059_2024_3435_MOESM1_ESM.docx]

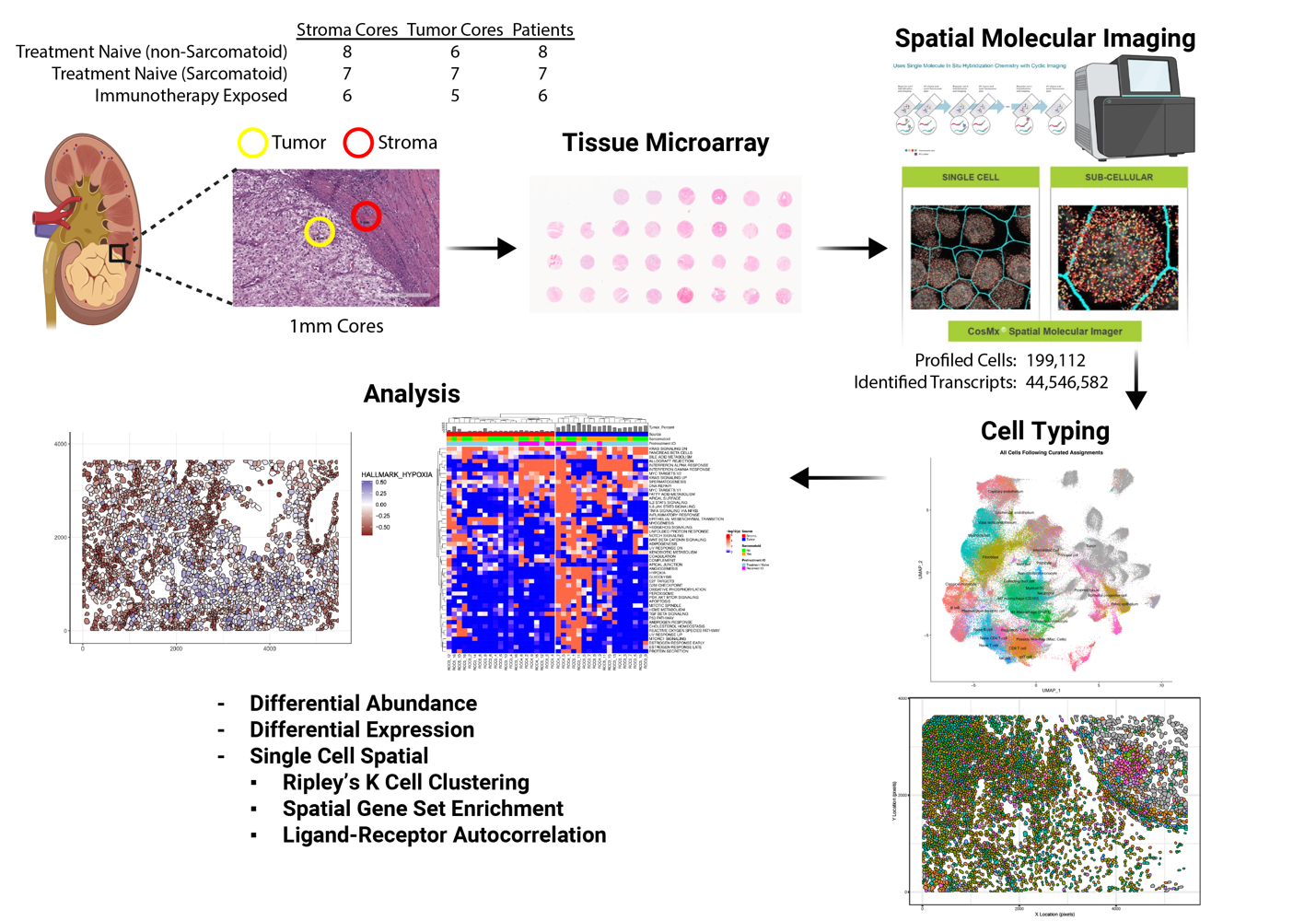


**Figure S1.** Study overview showing the number of samples per group, data generation with CosMx Spatial Molecular Imaging, cell-type identification, and analyses.


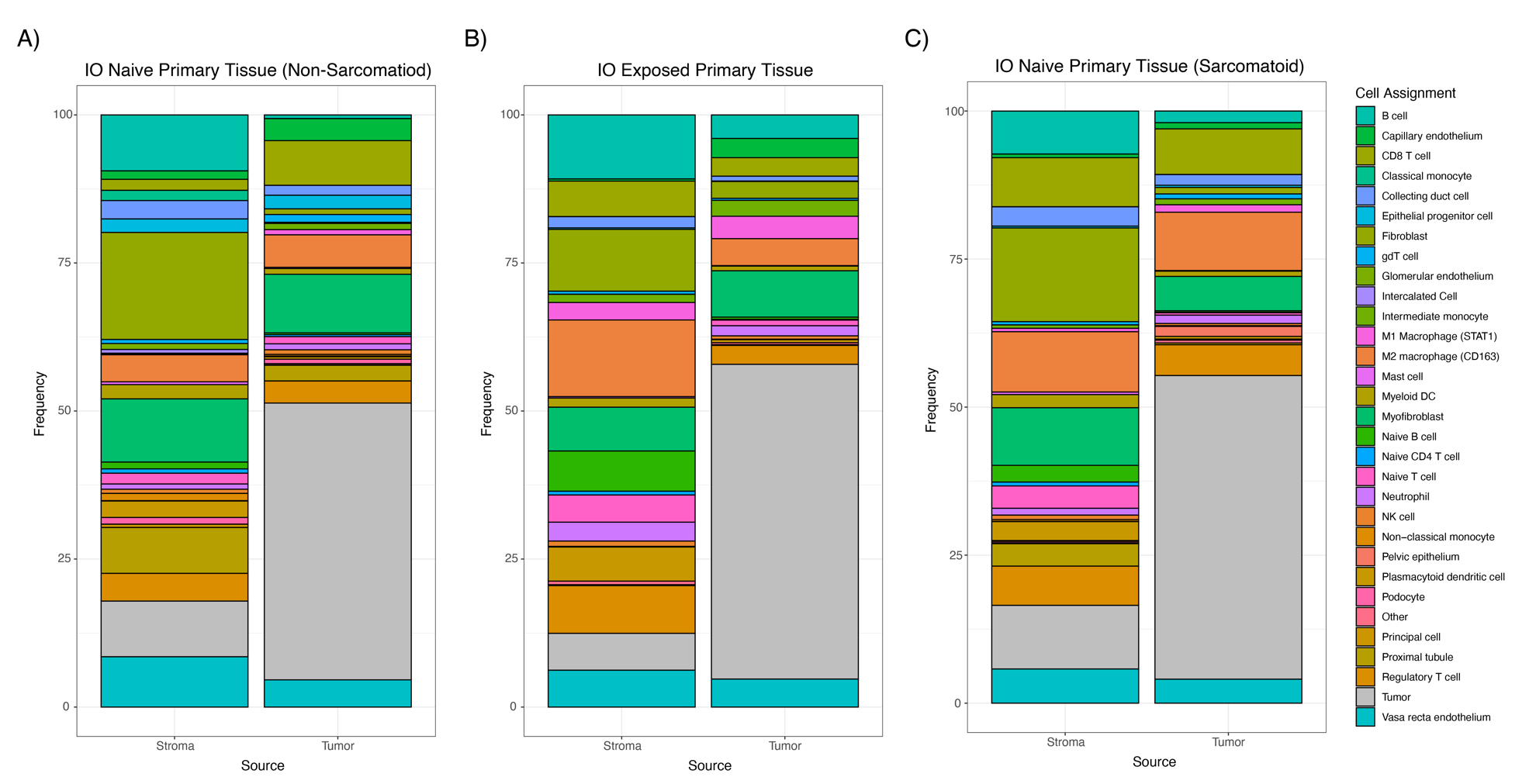


**Figure S2.** Abundance of final cell assignments. (A) shows IO naïve primary nonsarcomatoid; (B) shows IO exposed; and (C) shows IO naïve primary sarcomatoid stroma and tumor FOV.


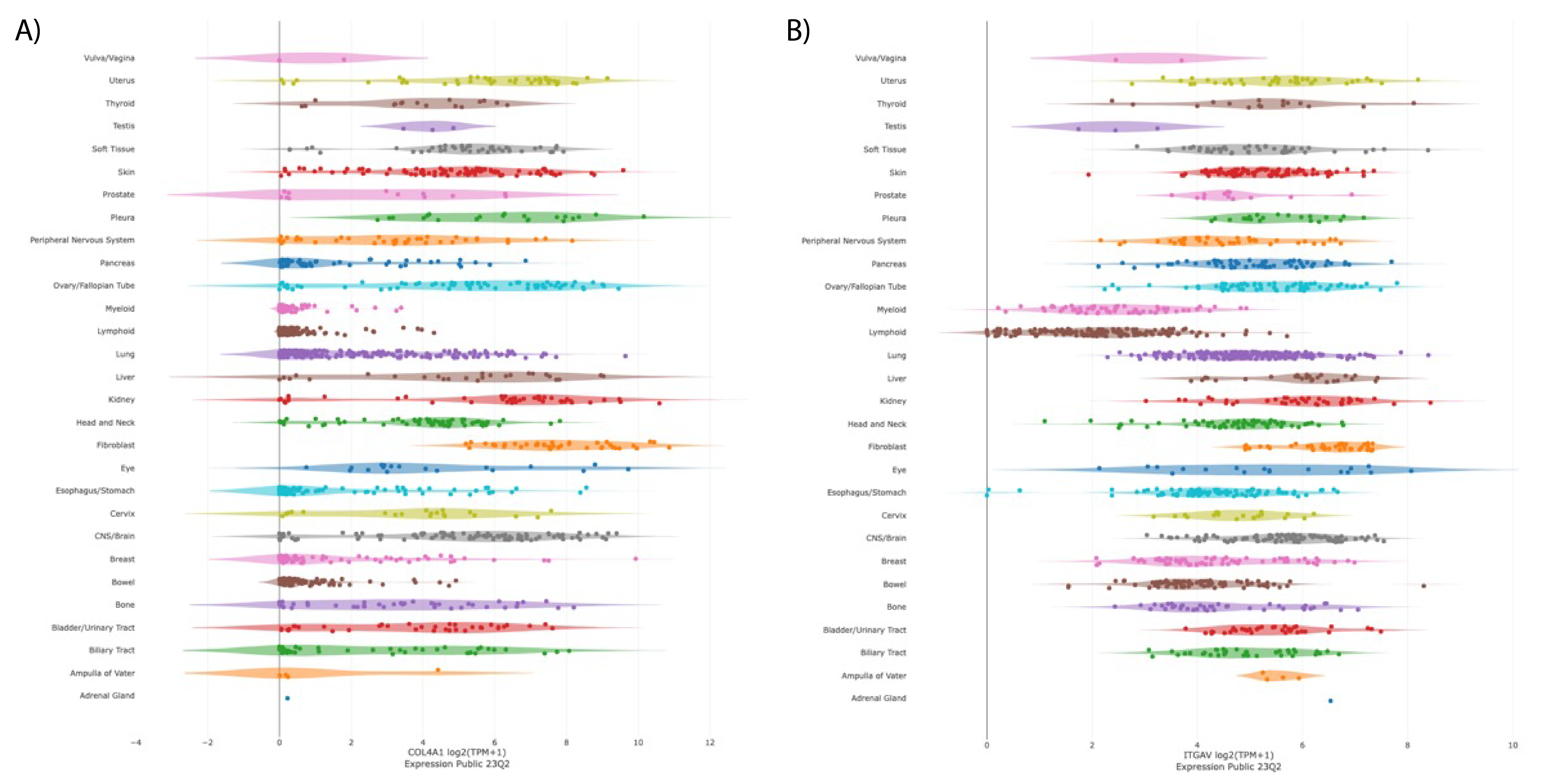


**Figure S3.** Expression of *COL4A1* and *ITGAV* in normal tissues from GTEx project. Abbreviation: GTEx, Genotype-Tissue Expression.
