# Supplementary material for: Increased spatial coupling of integrin and collagen IV in the immunoresistant clear-cell renal-cell carcinoma tumor microenvironment
Source: Genome Biol. 2024 Dec 5;25:308. doi: 10.1186/s13059-024-03435-z (PMC11622564; doi:10.1186/s13059-024-03435-z)
Supplement: Supplementary file 4 — Additional File 4. Review history. [file 13059_2024_3435_MOESM4_ESM.docx]

Review History

**First round of review**

**Reviewer 1**

**Were you able to assess all statistics in the manuscript, including the appropriateness of statistical tests used?**

Yes, and I have assessed the statistics in my report.

**Comments to author:**

In the manuscript, Soupir et al. present "Increased spatial coupling of integrin and collagen IV in the immunoresistant clear cell renal cell carcinoma tumor microenvironment." Overall, this is an important resource generated to help better understand immunotherapy resistance mechanisms in ccRCC. However, the authors should perform external validation on independent validation data sets for supporting their key conclusions. In addition, the manuscript requires more reorganization for clarity.

Major comments as followed.
1.The language should be improved systematically.
2.The author should include a schematic figure to illustrate the design of the study beside Table 1. It is very hard to get what kind of samples are used and what kind of techniques are applied in the current manuscript.
3.The subtitle of the result part should present with findings but not method description.
For example: "Subclustering T cells and Mononucleic Phagocytes (MNPs)" is not a result. Therefore, in Figure 1, it is hardly drawn any conclusion from it. There are plenty of published papers which applied scRNA-seq technology, the authors should provide more information on findings.
4.Figure 2, should re-organized. FOVs of tumor and stromal should be highlighted, and umap of tumor specific FOV and stromal-specific FOV should be present side by side. Meanwhile, please explain why there are principal cells in Figure 2F but not Figure 2A. The author should also use algorithms such as infercnv and/or identification by pathologist to confirm the cells from tumor-specific FOV are indeed tumor cells.
5.Figure 2C and 2H is under different scale, it was hardly to conclude the expression of VEGFA in higher in tumor cells than that of proximal tubule cells.
6.It is also very difficult to get the point from Figure 3. Supervised clustering may be more suitable to present the data. For example, comparing treatment naive and received IO; sarcomatoid and non-sarcomatoid in different panel.
7.In Figure 4, multiplex immunofluorescence staining Fibroblasts, myofibroblasts, endothelial cells, COL4A1 and ITGAV expression is required to get the conclusion.
In Figure 5, without single cell resolution, bulk analysis did not give any further insight.

**Reviewer 2**

**Were you able to assess all statistics in the manuscript, including the appropriateness of statistical tests used?**

Yes, and I have assessed the statistics in my report.

**Comments to author:**

Soupir et al. reported a spatial transcriptomic analysis of ccRCC samples (n=21) either exposed or not exposed to IO using NanoString CosMx technology. To do so, they selected one spot from the tumor and stroma and they analyzed using different algorithms cell types and pathways associated with sarcomatoid dedifferentiation or difference between IO exposed tumors or not. As a conclusion, they identified increased coupling of integrin and collagen IV in resistant ccRCC microenvironment. The study is novel in its types and its adding lots of information's towards our understanding of the disease. However, I have major concerns that needs to be addressed:

1) The authors used 1 mm spot for each of the tumors and stroma area per patient. How the authors can account for the heterogeneity of ccRCC as these tumors are highly heterogenous and we might expect variation between the center of the tumor and the interface with the normal
2) I am really surprised by the low number of genes (n=51) that can distinguish cancer from normal proximal tubules. This is could be somehow explained by the low number of probes (n=1000) of NanoString CosMx technology. However, if the difference between cancer and normal is so limited, the reviewer is wondering how this technology can be used to define cell states and the presence of EMT in these tumors. For instance, using Li et al. data (doi: 10.1016/j.ccell.2022.11.001), can the author comment on the added value of their spatial analysis to define cell types.
3) The authors identified one gene (YES1) which was significantly upregulated in IO exposed tumor cells. Is it really by chance? The reviewer understand that the technology allows only the analysis of limited genes, but how much we can be confident with only the expression of gene between responders and non-responders? I suggest for the authors to validate the data in public cohorts of patients treated with IO.

**Authors Response**

**Point-by-point responses to the reviewers’ comments:**

Reviewer #1: In the manuscript, Soupir et al. present "Increased spatial coupling of integrin and collagen IV in the immunoresistant clear cell renal cell carcinoma tumor microenvironment." Overall, this is an important resource generated to help better understand immunotherapy resistance mechanisms in ccRCC. However, the authors should perform external validation on independent validation data sets for supporting their key conclusions. In addition, the manuscript requires more reorganization for clarity.

Major comments as followed.

**1. The language should be improved systematically.**

We thank the reviewer for their suggestion of improving language throughout the manuscript. We have submitted the manuscript for review by MCC Office of Scientific Publishing, and the final resubmitted version has their edits incorporated throughout the manuscript.

**2. The author should include a schematic figure to illustrate the design of the study beside Table 1. It is very hard to get what kind of samples are used and what kind of techniques are applied in the current manuscript**.

We thank the reviewer for their suggestion of adding a schematic figure of the study overview and have added Supplementary Figure 1 (Figure 1 of this document). This figure includes our samples and the breakdown of tumor/stroma for each analysis group; the profiling of spatial transcriptomics; the cell-type identification; and the high-level analyses. We hope this makes the study easier to follow and comprehend.

**Figure 1.** Overview of the study including tumor locations, molecular profiling, cell phenotyping, and spatial analyses.

**3. The subtitle of the Results should present with findings but not method description.
For example: “Subclustering T cells and Mononucleic Phagocytes (MNPs)” is not a result. Therefore, in Figure 1, it is hardly drawn any conclusion from it. There are plenty of published papers which applied scRNA-seq technology, the authors should provide more information on findings.**

We thank the reviewer for their suggestion for correcting this subheading to reflect the findings in the Results section. Figure 1 displays the result of further cell type refinement performed by subclustering the broader T cell and MNP cells. We have updated the subheadings throughout the Results section in a similar manner. .

**4.** **Figure 2 should be reorganized. FOV of tumor and stromal should be highlighted, and UMAP of tumor-specific FOV and stromal-specific FOV should be presented side by side. Meanwhile, please explain why there are principal cells in Figure 2F but not 2A** .

**The author should also use algorithms such as infercnv and/or identification by a pathologist to confirm that the cells from tumor-specific FOV are indeed tumor cells.**

We thank the reviewer for the suggestion on **Figure 2**’s layout; placing the like-plots side by side would better compare the tumor and stroma FOV (**Figure 2**). We agree that this layout makes it easier to compare the differences between the kidney-specific cells in the 2 regions.

**Figure 2**. Restructured Figure 2.

For principal cells existing in **Figure 2F** (now **2B**) but not in **2A**, this is caused by no principal cells being identified through the *InSituType* cell annotations for FOV RCC3-1 (tumor-specific FOV). **Supplementary Figure 2** (previously **Supplementary Figure 1**) shows the abundance of the 31 different cell types identified across all FOV. The largest proportion of principal cells identified in any single FOV was 1.39%, and across the 39 FOVs, only 18 had at least 1 principal cell. A new abundance plot has been produced below (**Figure 3**), which focuses on principal cells.

**Figure 3.** Re-shaded **Supplementary Figure 1** from the manuscript to highlight the low abundance of annotated principal cells across all sample groups. This demonstrates why principal cells appear in **Figure 2b** but not **Figure 2a**.

Currently, there is not a gold-standard approach for identifying malignant cells in kidney cancer, so there is no dataset that is considered the ground truth to which we can compare the performance of our model. For the present study, we relied on the expertise of a genitourinary pathologist (JD), who reviewed our malignant-cell annotations alongside H&E images of adjacent sections to confirm that we were capturing the correct regions (mentioned in text). This provides a great benefit over scRNA data because we can leverage the pathologist’s expertise, which employs spatial and morphologic characteristics of the cells to identify regions of tumor vs surrounding stroma. Further, a few FOV (such as FOV 17 on slide RCC4) were selected/planned to be tumor FOV, but after tissue processing, application of our model showed very few malignant cells, and review by our pathologist resulted in reclassification to a stromal FOV before analysis.

*Infercnv* has not been validated on imaging-based spatial transcriptomic platforms. To explore *infercnv* on our data, we had to set the minimum counts per cell lower than the recommended 100 (to 18) because of the lower number of genes profiled compared to scrnaseq, resulting in a lower number of transcripts identified per cell. We also removed reference-group names because we do not know which cells (a priori) specifically are *normal* and *tumor*; we only know of mixes of cells in both the tumor FOV and stromal FOV per the pathologist’s review (hence possible reclassification based on abundance of malignant cells). This resulted in 25 of our 958 genes (1 gene in SMI not available in Ensembl biomaRt for genome locations needed in inverncv; 933 remaining) being removed because of the “taking into account provided gene ordering list” step, and 22 cells that had expression within these genes that now fall below the 18-transcript threshold set above. The next threshold used in infercnv::run is with a cutoff of 0.1, which again removes a further 535 genes, leaving 398 genes for Step 3 of infercnv. With the number of genes that CosMx SMI starts with, and the zero-inflated nature of the data, we do not think that the results are reliable (all cells belonged to the *tumor* group found in infercnv_object@observation_grouped_cell_indices and none in the normal infercnv_object@reference_grouped_cell_indices). Because of this, we believe ourapproach utilizing direct pathologic annotations to confirm malignancy is rationale, accepting all methods have potential limitations. .

Reviewer 2 also raised the concern about identifying cell states such as malignant vs normal proximal tubule cells based on the low differential gene expression in combination with the smaller panel. To answer the questions: (1) Are we able to correctly identify malignant cells? Which then also answers (2) Are cell states able to be identified with the CosMx SMI platform? We leveraged the public dataset from Li et al [1], which has malignant and normal labeled single-cell RNA-seq data. With this, we found that our LASSO model produced an area under the curve (AUC) of 0.92, and using a threshold of 0.5 accurately predicted 97.9% malignant cells in the external dataset. This confirmatory analysis has now been added to our manuscript.

**5. Figure 2C and 2H are under a different scale, so it was difficult to conclude the expression of VEGFA as higher in tumor cells than in proximal tubule cells.**

**Figure 4.** Expression of *VEGFA* in proximal tubule cells from a tumor (RCC3_1) and stromal (RCC3_2) FOV.

We would like to thank the reviewer for pointing out the difficulty interpreting the histograms of proximal tubule *VEGFA* expression in the tumor (RCC3-1) and stroma (RCC3-2) FOV. We have plotted the expression again for these 2 FOVs using the same scales, as well as emphasized the SCTransform value of 0 (**Figure 4**). Because of the difference in cell counts between samples, the histogram pile-up is not an exact comparison, but this does show a much larger distribution of expression of *VEGFA* in the tumor FOV, indicating malignant proximal tubule cells, compared to the stroma FOV, where very few proximal tubule cells have expression greater than SCT = 0. *VEGFA* expression in this tumor FOV ranged from −0.8635 to 9.8166, while in this stromal FOV ranged from −0.9715 to 3.3058.

**6. It is also very difficult to get the point of Figure 3**. **Supervised clustering may be more suitable to present the data. For example, comparing treatment naïve and received IO; sarcomatoid and non-sarcomatoid in different panel.**

Thank you for your suggestion to plot **Figure 3** differently to allow the results to be better understood. For completeness, we have reorganized the in-text figure, separating out the comparison groups along with the tissue source (**Figure 5**). For clarity in this response, we have removed the stroma and separated out the tumor comparison groups: IO-exposed/received IO tumors, treatment-naïve nonsarcomatoid tumors, and treatment-naïve sarcomatoid tumors. As better pictured here, it’s evident that tumors following IO exosure show significant spatial clustering (red in the heatmap; cells with high enrichment scores closer than random) across more Hallmark gene sets than tumors that are IO naïve.

**Figure 5.** Semisupervised clustering of tumor FOV to show significant spatial clustering of cells with high enrichment across gene sets.

**7. In Figure 4, multiplex immunofluorescence staining Fibroblasts, myofibroblasts, endothelial cells, COL4A1 and ITGAV expression is required to get the conclusion. In Figure 5, without single-cell resolution, bulk analysis did not give any further insight.**

We thank the reviewer for their comment. We believe you are referencing **Figure 5** (multiplex IF on orthogonal sections of the same TMA used for CosMx SMI profiling) instead of **Figure 4** (polygon locations of myofibroblasts and malignant tumor cells overlayed on top of smoothed *COL4A1* and *ITGAV* expression along with polygon locations of all cells in the sample and overview of VEGF, integrin, and YAP proteins are related). The **Figure 5** callout was missing from the text but is present in **Figure legends** section.

We generated a new figure showing the expression of SMA+ with/without ITGAV or COL4 in the multiplex immunofluorescence assay (**Figure 6** in this document). This figure displays the increase in SMA+ ITGAV+ (purple) in both the tumor and stroma FOV from IO-naïve to IO-exposed tumors.

We also explored other manuscripts that have since been published while ours was under review and found a niche-differential expression analysis in colorectal cancer metastasis to the liver [2]. Here, the authors examined ligand-receptor interactions, however, using different methodology. Genes found were also associated with fibroblasts and were in *crosstalk* with malignant tumor cells. Significant to our findings, the authors identified *COL4A1* showing enrichment in regions where tumor cells and fibroblasts were colocalized on 10× Visium samples. We contribute to the literature to more specifically associate these genes with cell types at the single-cell level rather than at the spot level of 10× Visium, as well as to add the receptor for which *COL4A1* is a ligand, *ITGAV*, showing significant spatial autocorrelation.

**Figure 6.** The abundance of cells positive for marker combinations on multiplex immunofluorescence in orthogonal TMA sections.

Reviewer 2: Soupir et al. reported a spatial transcriptomic analysis of ccRCC samples (n=21) either exposed or not exposed to IO using NanoString CosMx technology. To do so, they selected one spot from the tumor and stroma and they analyzed using different algorithms cell types and pathways associated with sarcomatoid dedifferentiation or difference between IO exposed tumors or not. As a conclusion, they identified increased coupling of integrin and collagen IV in resistant ccRCC microenvironment. The study is novel in its types and its adding lots of information's towards our understanding of the disease. However, I have major concerns that needs to be addressed:

**1. The authors used a 1-mm spot for each of the tumors and stroma area per patient. How the authors can account for the heterogeneity of ccRCC, as these tumors are highly heterogenous, and we might expect variation between the center of the tumor and the interface with the normal …**

We thank the reviewer for their concern regarding tumor heterogeneity being captured by 1-mm tissue microarray cores. We understand that it’s difficult to capture to totality of tumor variation with 1-mm cores. However, we elected for this study to focus on capturing a larger number of tumors to increase biological variance had stronger clinical value at this time. We do however agree that future, larger studies utilizing multiple and spatially distinct tumor samples per tumor will provide valuable molecular insights on how the TIME changes across a tumor. We hope to perform studies that address these issues in our follow up studies. . We think that our data and study provide a strong, novel perspective on gene-expression differences between tumors that have and have not been exposed to immunotherapy (both with and without sarcomatoid features). While in this study, we focus on a narrow facet that identified changes in *YES1* expression in tumor cells, spatial clustering of cells with high gene-set enrichment, and an increased positive spatial relationship (autocorrelation) of the ligand-receptor pair *COL4A1*-*ITGAV* in samples following exposure to immunotherapy, the dataset being made public is a big resource for other researchers to data mine. Further, other researchers would be more able to contribute a slide with 20+ tissue microarray cores for a cohort of interest and to compare to our dataset.

**2. I am really surprised by the low number of genes (n = 51) that can distinguish cancer from normal proximal tubules. This could be somehow explained by the low number of probes (n = 1000) of NanoString CosMx technology. However, if the difference between cancer and normal is so limited, the reviewer is wondering how this technology can be used to define cell states and the presence of EMT in these tumors. For instance, using Li et al. data (doi: 10.1016/j.ccell.2022.11.001), can the author comment on the added value of their spatial analysis to define cell types.**

We thank the reviewer for their comment about the number of genes identified between malignant and normal proximal tubule cells. We would like to point out that while 1000 probes (959 genes in our data or 960 genes passing QC) is a fraction of the whole transcriptome (>18 000 for scRNA-seq), the nature of CosMx SMI is also more zero-inflated. With the genes that were selected for the CosMx SMI panel by Nanostring were selected for their ability to help with differentiating cells and cell state, 51 genes between cells of the same kind (proximal tubule) is slightly more than 5% of genes showing significantly different expression profiles.

Additionally, we created a classifier to determine whether or not kidney cells were malignant. Our pathologist (JD) compared H&E figures with the predicted malignant cell locations (polygon plots) and agreed to the consensus that the predicted cells were in fact malignant. To further validate this, we acquired the suggested single-cell data from Li et al [1]. “SCTransform” was applied across raw UMI counts of all 272,863 cells in the dataset. “RCC tumor cells,” “Endothelial cells,” “Proximal tubule epithelial cells,” and “nonproximal tubule epithelial cells” were selected for testing. Transformed scRNAseq expression of 42 out of 43 genes in the LASSO model were present, except *WIF1*. After discussion with the senior author of Li et al., the exclusion of *WIF1* is likely a reflection of sparsity in the scRNAseq dataset, as the 10X Chromium Platform library is known to be comprehensive with *WIF1* present in other studies [3]. Thus, imputation of 0 was used for *WIF1* expression for all cells. The pretrained regression model was applied to this expression data and compared to the true RCC labels in the dataset.

We found that 13,105 tumor cells, 4,597 endothelial cells, and 4,357 epithelial cells were available (22 059 kidney *tissue* cells) from the data. Applying our LASSO classification model, we obtained an AUC of 0.91 (**Figure 7**):

**Figure 7.** Receiver-operating characteristic curve for malignant cell classification in Li et al single-cell RNA-seq data.

Further exploring the assigned probabilities of a cell being malignant, we found that the model predicted tumor cells as being malignant 97.9% of the time (12,833 predicted of the 13,105 tumor cells) with a threshold of 0.5—the same as the CosMx SMI. The model also performed moderately well in identifying cells that were not malignant in the new, independent dataset, predicting 69.5% of nonmalignant cells correctly (6,226 predicted of 8,954 other tissue cells).

We also tested a recently published tool in *Nature Communications* trained across 13 different cancer types to pick out malignant cells (**Table 1**; kidney not included in model training, [4]). The authors used 4,752 and 5,000 genes as features for their deep-learning models (our CosMx has 959 genes that passed all QC). The models suggested using the default threshold of 0.5 for scRNA, Visium, “legacy ST” and slide-seq, and using 0.9766 for MERFISH. We used the recommended 0.5 and 0.9766 and noticed that both thresholds were greatly undercalling tumor cells; see table below. Lowering the threshold of the ST model to 0.25 increased the number of cells being identified as “tumor” but was still far below what the LASSO model predicted in our samples, while also calling varying proportions of other cell types as malignant. As noted, the limitations of this method include both the gene coverage of lower-plexed imaging-based spatial transcriptomics that results in missing features in the model, as well as kidney data being removed from the training because malignant cells were not present.

**Table 1.** Number of cells predicted to be tumor/malignant with different thresholds of Zhong et al deep-learning Cancer Finder model.

| Predicted Tumor | sc 0.5 | sc 0.9766 | st 0.25 | st 0.5 | st 0.9766 | LASSO |
| --- | --- | --- | --- | --- | --- | --- |
| LASSO Cells | 1892 | 0 | 17978 | 2216 | 0 | 38026 |
| All Cells | 2214 | 0 | 23841 | 2306 | 0 | 38026 |

These results indicate the strong ability of this technology (CosMx SMI) to identify cell types and states, and that models developed on the CosMx SMI platform can be applied with reasonable accuracy to external datasets of differing technology.

For the epithelial mesenchymal transition (EMT) Hallmark gene set, there are a total of 200 genes, and 75 of those are included in the CosMx SMI panel. Studies have shown that tools created for bulk sequence analysis perform well on single-cell data, even sometimes favorably to tools developed for single-cell data [5]. EMT has long been noted to be associated with advancing disease in many cancers [6]. Not only did we use the enrichment scores for genes sets like EMT, but we leveraged the spatial locations of them as well. If we were not able to pick up any signal with enrichment scores, we would expect spatial enrichment to identify significant clustering of highly enriched cells at the false-positive rate for the test (i.e. randomly in the data regardless of the treatment/histology features). Since we *did* identify changes between sample groups (ie, IO naïve vs IO exposed) that do not appear to be random (consistently higher spatial enrichment following exposure to IO), we concluded that CosMx SMI is able to pick up differences in Hallmark gene-set states.

**3. The authors identified one gene (YES1) which was significantly upregulated in IO-exposed tumor cells. Is it really by chance? The reviewer understand that the technology allows only the analysis of limited genes, but how much we can be confident with only the expression of gene between responders and non-responders? I suggest for the authors to validate the data in public cohorts of patients treated with IO.**

We thank the reviewer for requesting external validation of the *YES1* finding, where it was significantly higher in malignant cells after their exposure to immunotherapy. Because of the lack of public spatial transcriptomic data/CosMx SMI data before and after immunotherapy exposure in ccRCC, we explored other malignancies to find an ability to assess changes to *YES1* expression in malignant cells, specifically as a result of exposure to immunotherapy. We identified the single-cell RNA-seq dataset GSE115978 with malignant (Mal) cells from IO-naïve and IO-exposed tumors [7]. Using the cell annotations from the Gene Expression Omnibus provided by the authors, there are 2018 malignant cells, with 1193 being from IO-naïve tumors and 825 from IO-exposed tumors.

**Figure 8.** Expression of *YES1* in malignant cells from a melanoma before and after exposure to an immune checkpoint inhibitor.

Plotting the expression of *YES1* in their IO naïve and IO exposed shows that the median of *YES1* log2 expression (red dot) in malignant cells trends higher after IO exposure (3; raw count = 7) compared to IO naïve (1; raw count = 1; **Figure 8**). Performing a Wilcoxon rank-sum test on the expression between IO exposure groups shows a *P* value of 1.08e-32, indicating a significant change in *YES1* log2 expression.

**References**

1.	Li, R., et al., *Mapping single-cell transcriptomes in the intra-tumoral and associated territories of kidney cancer.* Cancer Cell, 2022. **40**(12): p. 1583-1599.e10.

2.	Mason, K., et al., *Niche-DE: niche-differential gene expression analysis in spatial transcriptomics data identifies context-dependent cell-cell interactions.* Genome Biology, 2024. **25**(1): p. 14.

3.	Farbehi, N., et al., *Single-cell expression profiling reveals dynamic flux of cardiac stromal, vascular and immune cells in health and injury.* Elife, 2019. **8**.

4.	Zhong, Z., et al., *Domain generalization enables general cancer cell annotation in single-cell and spatial transcriptomics.* Nature Communications, 2024. **15**(1): p. 1929.

5.	Holland, C.H., et al., *Robustness and applicability of transcription factor and pathway analysis tools on single-cell RNA-seq data.* Genome Biol, 2020. **21**(1): p. 36.

6.	Smith, B.N. and N.A. Bhowmick, *Role of EMT in Metastasis and Therapy Resistance.* J Clin Med, 2016. **5**(2).

7.	Jerby-Arnon, L., et al., *A Cancer Cell Program Promotes T Cell Exclusion and Resistance to Checkpoint Blockade.* Cell, 2018. **175**(4): p. 984-997.e24.

**Second round of review**

**Reviewer 1**

The manuscript has been systematically improved in case of clarity. There are still several comments should be addressed.

1.In the conclusion part of abstract: . “Integrin represents a potential therapeutic target in IO treated ccRCC. “ The conclusion is over-addressed which can not support by current evidences. The description as the discussion part “Additional study is needed to elucidate the biological basis for this shift in the ccRCC TIME, as well as to examine the possible therapeutic potential of integrin following IO treatment.” is appropriate .
2.In the cell phenotyping in Figure 2, please make sure the reference geneset used to identify “epithelial progenitor cell” since it was not usually identify in scRNA-seq data from kidney tissue.
3.Figure 4 is much clearer in the revised manuscript. However, I would suggest the authors can highlight or drive new plot to present the major conclusions.
4.In Figure 5, I suggest move the Supplementary Figure 3 to main Figure 5. Meanwhile, the cell assignment bar is for Figure 5B? should be labeled more clearly. Meanwhile, since the major conclusion of the manuscript is largely from myofibroblast, fibroblast, endothelial cell and tumor cells, I will suggest used color code will significantly distinguish the above cell types. For example, in the manuscript, capillary endothelium, collecting duct cell, epithelial progenitor cells, fibrablast and myofibroblast are all in green. It is very hard to directly draw the conclusion.
5.In Figure 6, the authors can integrate and present the results of Supplementary Table 13 and 14, and include the result in main Figure.

**Reviewer 2**

The authors answered all my concerns
They have to be congratulated on the amounts of work done

**Authors Response**

**Point-by-point responses to Reviewer #1’s comments:**

The manuscript has been systematically improved in case of clarity. There are still several comments should be addressed.

1.In the conclusion part of abstract: . “Integrin represents a potential therapeutic target in IO treated ccRCC. “ The conclusion is over-addressed which can not support by current evidences. The description as the discussion part “Additional study is needed to elucidate the biological basis for this shift in the ccRCC TIME, as well as to examine the possible therapeutic potential of integrin following IO treatment.” is appropriate .

*Thank you for the suggestion to remove the over-interpretation of the findings. I have changed the text within the conclusions section of the abstract to remove the overstatement of findings to stick with main findings (“Spatial transcriptomics of samples of 3 patient cohorts with cRCC tumors indicates that COL4A1 and ITGAV are more autocorrelated in immunotherapy-exposed stroma compared to immunotherapy-naïve tumors, with high expression amongst fibroblasts, tumor cells, and endothelium”) and stated “Further research is needed to understand changes in the ccRCC tumor immune microenvironment and explore potential therapeutic role of integrin after immunotherapy treatment.”*

2.In the cell phenotyping in Figure 2, please make sure the reference geneset used to identify “epithelial progenitor cell” since it was not usually identify in scRNA-seq data from kidney tissue.

*Thank you for the comment. Cell phenotyping is described within the methods section of the manuscript, where the Kidney Cell Atlas was used to create a signature matrix that was then provided to InSituType for cell typing. Of the Kidney Cell Atlas cell types was ‘epithelial progenitor cell’:* [*https://www.kidneycellatlas.org/mature-kidney-global*](https://urldefense.com/v3/__https:/www.kidneycellatlas.org/mature-kidney-global__;!!NLFGqXoFfo8MMQ!p4A2pA2uxN7QlQHwzq8w_hqG_pbKhXDbM-kCb_0YpKsQQ9-UpldsSGNpaXNKnwTxQOC92F2C9uP-I_cI6f81rp8rbDy2KewEIg$) *see ‘celltype’. Text was added to clarify that the Kidney Cell Atlas was used.*

3.Figure 4 is much clearer in the revised manuscript. However, I would suggest the authors can highlight or drive new plot to present the major conclusions.

*Thank you for the suggestion. We believe the increase in significant spatial gene set enrichment from left to right while being sorted by treatment and tissue location is depicted strongly, especially in the case of “Received IO – Tumor” samples, and follows the text where stated “This is displayed in Figure 4 in ‘Tumor Locations’ with increasingly significant spatial clustering, moving from Treatment Naïve (left) to Sarcomatoid (middle) to finally those tumors that were IO-exposed (right).*

4.In Figure 5, I suggest move the Supplementary Figure 3 to main Figure 5. Meanwhile, the cell assignment bar is for Figure 5B? should be labeled more clearly. Meanwhile, since the major conclusion of the manuscript is largely from myofibroblast, fibroblast, endothelial cell and tumor cells, I will suggest used color code will significantly distinguish the above cell types. For example, in the manuscript, capillary endothelium, collecting duct cell, epithelial progenitor cells, fibrablast and myofibroblast are all in green. It is very hard to directly draw the conclusion.

*Thank you for the suggestion of moving Supplementary Figure 3 to be part of main Figure 5. I have moved it to be part of Figure 5 as subpanel C. To address the colors used for distinguishing between cell types, I have muted the other cell types that are not of significant interest to make fibroblasts, myofibroblasts, and the malignant tumor cells stand out in subpanel B while also converting to the color-blind friendly palate. To make sure the colors for each cell type are maintained across the manuscript, I have stuck with these 3 cell types as they were of larger focus as well as decreases the number of different colors that are present at full opacity.*

5.In Figure 6, the authors can integrate and present the results of Supplementary Table 13 and 14, and include the result in main Figure.

*Thank you for the suggestion for incorporating Supplementary Tables 13 and 14 into the main Figure 6. I have added a simplified version of these tables to the figure as subpanels C and D while maintaining the full details in the Supplementary Tables which are now 14 and 15 after moving main Table 2 to Supplementary Table 11 due to being rather large.*
